# High-resolution taxonomic profiling and metatranscriptomics identify microbial, biochemical, host, and ecological factors in peri-implant disease

**DOI:** 10.1128/msystems.00754-25

**Published:** 2026-07-31

**Authors:** Szymon P. Szafrański, Amruta A. Joshi, Matthias Steglich, Ines Yang, Taoran Qu, Wiebke Behrens, Uthayakumar Muthukumarasamy, Damianos Melidis, Paula Schaefer-Dreyer, Jasmin Grischke, Jan Hegermann, Wolfgang Nejdl, Susanne Häussler, Meike Stiesch

**Affiliations:** 1 Department of Prosthetic Dentistry and Biomedical Materials Science, Hannover Medical School 9177, Hanover, Germany; 2 Lower Saxony Centre for Biomedical Engineering, Implant Research and Development (NIFE), Hannover, Germany; 3 Cluster of Excellence RESIST (EXC 2155), Hannover Medical School 9177, Hanover, Germany; 4 Department of Molecular Bacteriology, Helmholtz Centre for Infection Research 28336, Braunschweig, Germany; 5 L3S Research Center, Leibniz University Hannover 26555, Hanover, Germany; 6 Research Core Unit Electron Microscopy, Institute of Functional and Applied Anatomy, Hannover Medical School 9177, Hanover, Germany; 7 Knowledge-based Systems Laboratory, Leibniz University Hannover 26555, Hanover, Germany; 8 Institute for Molecular Bacteriology, Twincore, Centre for Clinical and Experimental Infection Research, Hanover, Germany; 9 Department of Clinical Microbiology, Copenhagen University Hospital-Rigshospitalet 683301, Copenhagen, Denmark; Medical Research Council Toxicology Unit, Cambridge, United Kingdom

**Keywords:** oral microbiome, implanted devices, metatranscriptomics, microbial ecology, biofilms, bacteriophages, host response, metataxonomics

## Abstract

Biofilm-associated diseases like peri-implant mucositis (PIM) and peri-implantitis (PI) are significant clinical challenges affecting millions of dental implant patients globally. Although studies have described the role of microbial, host, or environmental factors in disease development, their complex interplay, particularly during dysbiosis, remains poorly understood. This cross-sectional study characterized the microbiome composition and metatranscriptomes of 125 peri-implant biofilms from 48 individuals, uncovering molecular signatures linked to peri-implant health (PIH), PIM, and PI. Distinct variations were observed in biofilm amount, microbial composition and activity, phage populations, and host response. Biofilms were categorized into four community types (CTs) based on the bacterial transcriptional activity: one linked to PIH, one to PI, and two to PIM. PIH and PIM were primarily characterized by aerotolerant taxa with increased anabolic processes, while PI was dominated by obligate anaerobes with complex biofilm morphology. PIM samples, relative to PIH, were characterized by biofilm expansion with minimal functional changes, except for the *Neisseria*-rich PIM subtype showing higher pyruvate and lipoic acid metabolism. The phagome mirrored the bacterial compositional variations across disease states. Furthermore, human transcriptome responses varied, indicating increased keratinization in PIH, enhanced expression of ribosome components in PIM, and inflammatory signaling and hypoxia in PI. Additionally, we identified complex species–enzyme, phage–bacterium, and host–microbe associations within the peri-implant ecosystem. Our integrative multi-omics approach provides a comprehensive view of microbial, biochemical, host, and ecological factors associated with dysbiosis, offering novel insights into peri-implant disease dynamics.

**IMPORTANCE:**

Peri-implant mucositis and peri-implantitis are highly prevalent inflammatory conditions that compromise the long-term survival and success of dental implants, yet their underlying biological mechanisms are largely unresolved. The full-length 16S rRNA gene amplicon sequencing (full-16S) allows for high-resolution taxonomic profiling of peri-implant biofilms, thereby advancing our understanding of microbial composition across health and peri-implant diseases. The integration of metatranscriptomics, furthermore, captures actively transcribed genes within the biofilm and offers direct insights into microbial community functions and the broader molecular context of peri-implant dysbiosis. DNA- and RNA-derived abundances were strongly correlated, with only a few microbial classes showing moderate diagnosis-related differences after DNA-based normalization of transcriptional activity. In this study, we integrated full-16S with metatranscriptomic profiling to simultaneously assess microbial taxonomy, functional activity, phage dynamics, and host gene expression in peri-implant biofilms. Importantly, we provide a systems-level view and report previously undescribed associations between different molecular signatures in the peri-implant ecosystem.

## INTRODUCTION

Millions of patients have received dental implants as an effective solution for tooth replacement (1). However, the growing number of dental implant placements has been accompanied by a rise in biofilm-associated peri-implant diseases. Peri-implant mucositis (PIM), a reversible infection of the peri-implant mucosa, has a prevalence of 43%, while peri-implantitis (PI), which involves progressive loss of the supporting bone, affects more than 25% of implants within 5 years of placement (2, 3). These peri-implant diseases impact patients’ quality of life and pose a substantial public health burden (4). The primary etiological factor for both PIM and PI is an imbalance within the microbial community characterized by uncontrolled biofilm expansion and the emergence of more virulent species (5–14). This dysbiosis triggers a host immune response which, if unsuccessful in clearing the infection, becomes dysregulated (8, 15, 16). The resulting immune dysfunction leads to tissue destruction, creating an environment that further promotes dysbiosis. Traditionally, peri-implant disease has been considered closely related to periodontal disease; however, emerging evidence suggests that the two ecosystems differ significantly (5, 8, 14, 17). While inter-subject variability in the submucosal microbiome has been observed among individuals with peri-implant disease, it remains unclear whether distinct community types (CTs), similar to those identified in periodontal disease, exist (18, 19).

Despite advances in molecular methods, the mechanisms underlying the development of the complex, multifactorial peri-implant conditions remain to be elucidated (8, 20). Metataxonomics and metagenomics overcame the limitations of early culture-based and DNA hybridization methods and enabled comprehensive profiling of the peri-implant microbiome and its functional potential (7, 11, 12, 14). However, short-read 16S sequencing lacks high resolution of taxonomic diversity and falls short in providing direct insights into bacterial activities, which can be uncovered by metatranscriptomics (MTX). Additionally, MTX provides a unique perspective on interspecies interactions, enabling predictions about their role in ecosystem dynamics and the development of dysbiosis (21, 22). Interestingly, MTX sequences from peri-implant biofilms also contain host and bacteriophage RNA, offering important insights into host–biofilm and phage–bacterium interactions (23, 24). However, technical challenges have limited the application of MTX on peri-implant biofilms, confining previous studies to two small patient cohorts (5, 16).

A multi-omics approach, integrating full-length 16S rRNA gene amplicon sequencing (full-16S) and MTX, represents a promising research direction for characterizing biofilm interactions and revealing the underlying mechanisms of microbiome dynamics and disease development (25–28), especially considering the complex and multi-dimensional nature of the submucosal ecosystem (22). In this study, we employed paired full-16S and MTX in the largest cohort of dental implant patients to date, aiming to advance a systems-level understanding of the peri-implant disease etiology. This integrative approach enabled a comprehensive characterization of the peri-implant ecosystem across three diagnostic groups, including the composition and activity of bacterial species and their community types, bacteriophages, enzymes, host genes, as well as key interactions among these components.

## RESULTS

**Fig 1:**
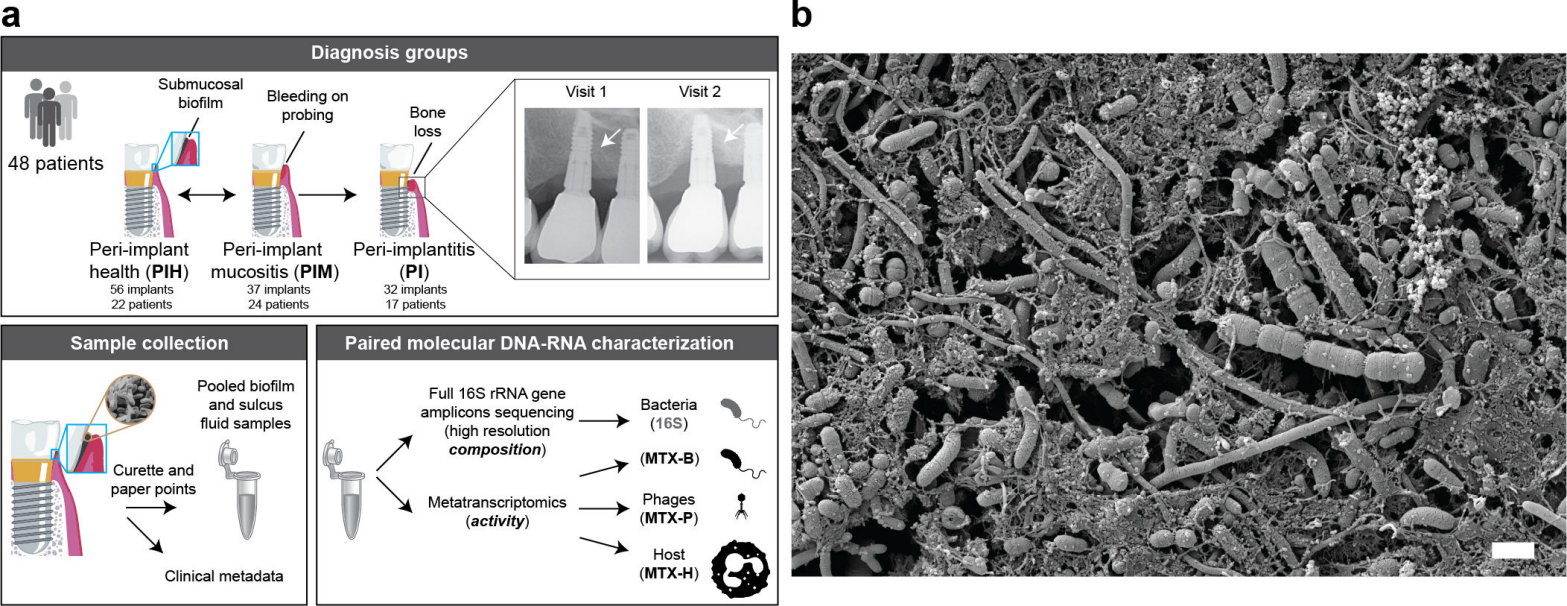
Study design. (**a**) Peri-implant diseases (top). Bone loss is an indicator of peri-implantitis. Radiographs showing crestal bone level in a representative clinical case with developing peri-implant disease. Collection of clinical samples (bottom left). Characterization of clinical samples (bottom right). For each implant, DNA- and RNA-based analyses were performed on the different nucleotide fractions of each sample. B-MTX, P-MTX, and H-MTX analyses were performed on the same sulcus fluid samples. (**b**) Scanning electron micrograph of a submucosal implant-associated biofilm scraped with a curette from an implant diagnosed with peri-implantitis. Length of scale bar: 1 µm. Panel **a** was adapted from Zheng et al. (30) under the Creative Commons Attribution 4.0 International (CC BY 4.0) license.

In this study, we analyzed the peri-implant microbiota composition and activity, including bacteriophages, and patient transcriptional responses from 125 implants in 48 patients (Fig. 1a). Peri-implant tissue condition was clinically classified as peri-implant health (PIH; *n* = 56), peri-implant mucositis (PIM; *n* = 37), or peri-implantitis (PI; *n* = 32) according to the “Classification of Periodontal and Peri-Implant Diseases and Conditions” (29). PI-associated implants showed significantly higher plaque index scores, poorer oral hygiene, greater probing depths, suppuration, and a history of periodontal disease (Table S1). PI was characterized by a higher incidence of pus formation and pain.

We obtained paired full-length 16S rRNA gene amplicon profiles (full-16S) and MTX for 125 implants. Full-16S was chosen over shotgun metagenomics because peri-implant biofilm samples, particularly from healthy sites, yield extremely low biomass, and our RNA-focused co-isolation protocol prioritizes RNA integrity at the expense of DNA quality (31). This strategy enabled high-resolution taxonomic profiling from the same RNA co-isolated samples used for MTX, thereby minimizing batch effects associated with independent sampling. In addition, it permitted DNA-based normalization of MTX profiles, facilitating the identification of taxa with elevated transcriptional activity relative to their genomic abundance. Twenty-nine out of 48 patients presented multiple implants (3.7 ± 1.9 implants; mean ± SD), which were all sampled to capture interpatient and intrapatient variability, while profiles were averaged by diagnosis during statistical testing to control for patient effects (32). MTX data concurrently characterized bacterial activity (MTX-B), phage activity (MTX-P), and host response (MTX-H). For MTX-B, we applied three complementary activity metrics: (i) biofilm-standardized transcriptional activity defined as relative transcript abundance within each sample (also used for MTX-P and MTX-H), (ii) abundance-normalized activity based on paired DNA-RNA data [log_2_(RNA/DNA)], and (iii) taxon-normalized activity, capturing within-taxon functional allocation. Sequencing details are provided in Table S2.

### Peri-implantitis exhibits dominance of anaerobic taxa, including many unnamed fastidious species

**Fig 2:**
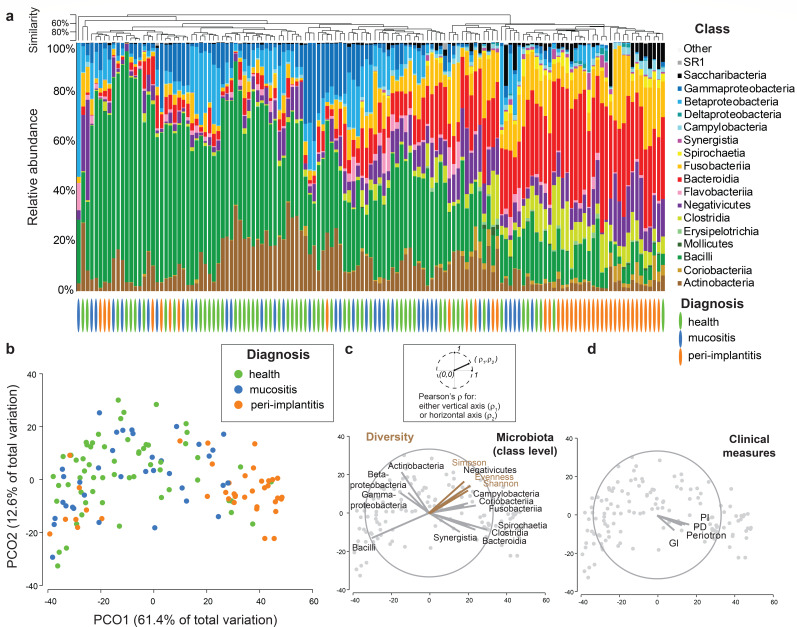
Composition of implant-associated biofilms at class level. (**a**) Composition of the 125 biofilms in 48 individuals was assessed by full 16S rRNA gene amplicon sequencing. Biofilms were ordered by hierarchical clustering of Bray–Curtis similarity values calculated for non-transformed standardized class abundance profiles. Classes were grouped by phyla. (**b**) Principal coordinates analysis (PCoA) of biofilm communities calculated on data from panel **a**. (**c**) Relationships between classes and diversity indices and ordination axes. (**d**) Relationships between clinical measures and ordination axes. Vector overlays in panels **c** and **d** were superimposed on the ordination from panel **b**. PD, probing depth; Periotron, measurement of peri-implant crevicular fluid volume; GI, gingival index; and PI, plaque index.

To identify the relative abundances of taxa that are differentially associated with PIH, PIM, and PI, we analyzed the composition of peri-implant communities (Table S3). Major compositional differences occurred at the class level between disease states (Fig. 2a and b, PERMANOVA pseudo-*F* = 3.15, *P* = 0.0028). PIH and PIM were generally characterized by a high abundance of aerobic or aerotolerant microorganisms of Bacilli, Actinobacteria, and Gammaproteobacteria, while PI showed dominance of strict anaerobes represented by Bacteroidia, Clostridia, and Deltaproteobacteria (Kruskal–Wallis adj. *P* ≤ 0.05). The difference between aerotolerant microorganisms in PIH and PIM and strict anaerobes in PI was confirmed using the log_2_(anaerobic/aerotolerant) ratio of aggregated read abundances (linear mixed-effects model with Tukey-adjusted pairwise comparisons: PIH vs PI, *P* = 0.001; PIM vs PI, *P* = 0.001). The biofilm composition in PIM was intermediate between that of PIH and PI, more closely resembling PIH (PERMANOVA *P* = 0.65 versus *P* = 0.01 for the PIM-PI comparison). Abundances of PI-associated classes positively correlated with clinical indicators of disease severity (Fig. 2c and d). Few outlier samples with taxonomic profiles not typical for a given diagnosis group were observed.

**Fig 3:**
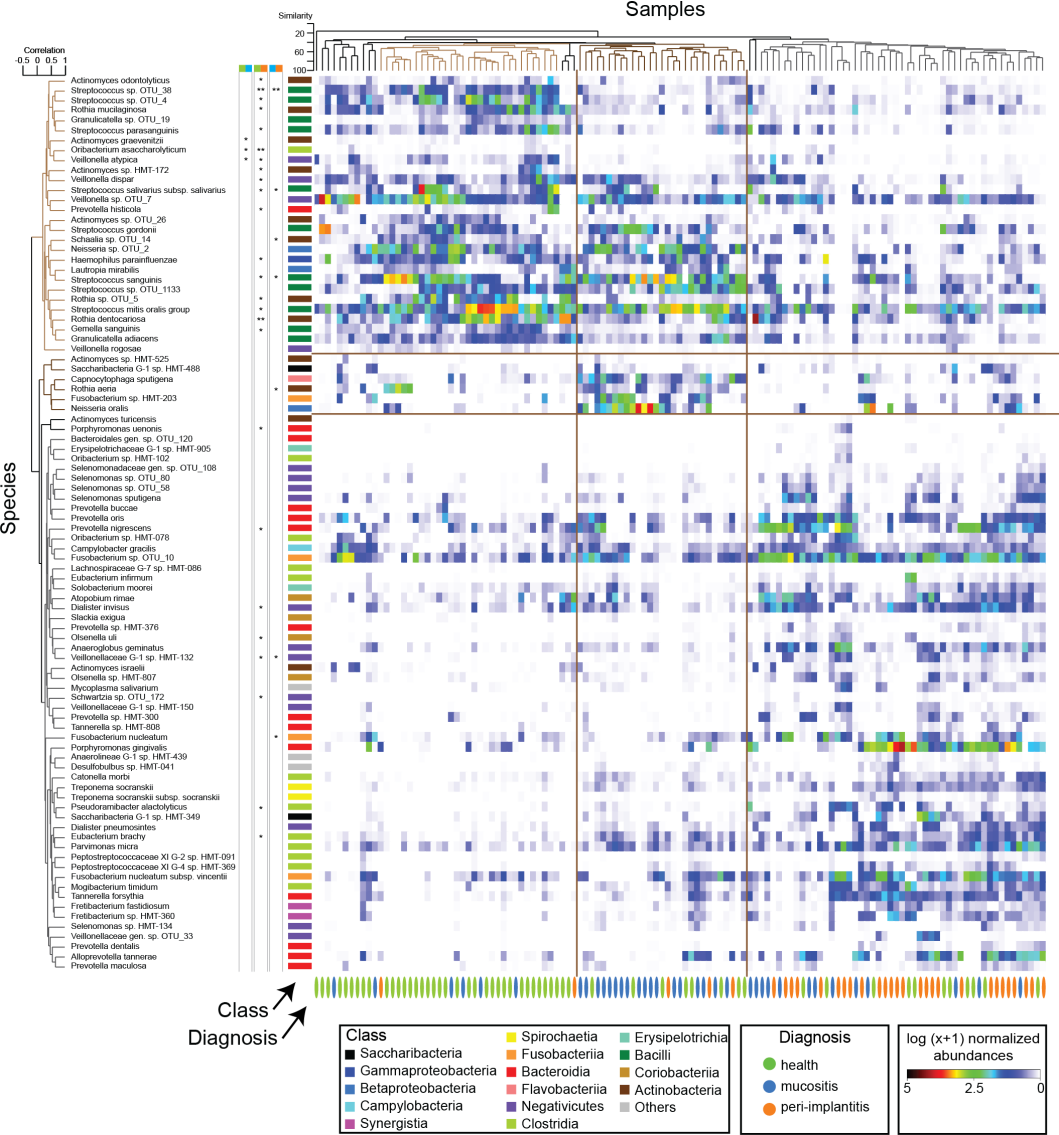
High-resolution composition of implant-associated biofilms. Heatmap indicating relative abundances of the top species-level taxa selected by canonical analysis of principal coordinates (CAP) analysis (Pearson | *r* | >0.4) across diagnosis groups. Horizontal colored bars indicate bacterial class, vertical bars below heatmap indicate the diagnosis associated with the sample. Samples ordered by hierarchical clustering of Bray–Curtis similarity values calculated for log(*x* + 1) transformed standardized species abundance profiles. Species ordered by hierarchical clustering of Spearman rank correlation values calculated for log(*x* + 1) transformed standardized species abundance profiles. Significant *P-*values for Mann–Whitney *U* test for pair-wise comparisons and Benjamini–Hochberg correction across diagnosis groups. ***P ≤ 0.0001; **P ≤ 0.001; *P ≤ 0.05.

At lower taxonomic levels, we identified a total of 99 different genera and 756 species-level phylotypes (Fig. 3; Tables S4 and S5). Microbial diversity, as reflected by the Shannon diversity index (H′), was higher in PI compared to PIH (3.4 ± 0.5 vs 3.1 ± 0.5, mean ± SD, Kruskal–Wallis, followed by *post hoc* Dunn’s test *P* = 0.02). In total, 23 genera and 28 species were dominant (average relative abundance of ≥1% in at least one diagnosis group), 30 genera and 34 species were core (present in ≥75% of samples in at least one group), and 13 genera and 63 species were associated with different diagnosis groups (Kruskal–Wallis FDR-corrected *P* < 0.05; Tables S4 and S5). At the genus level, *Streptococcus* was dominant and core in both PIH and PIM. PIM showed significantly higher abundances of *Shuttleworthia*, while *Aggregatibacter* and *Peptostreptococcus* were less abundant compared to PIH. PI showed expansion of *Dialister*, *Mogibacterium*, *Prevotella*, *Parvimonas*, *Pseudoramibacter*, and *Eubacterium,* outgrowing *Streptococcus* and *Rothia* when compared to PIM, and additionally, *Haemophilus*, *Actinomyces,* and *Aggregatibacter* when compared to PIH. At the species level, abundant species formed three major clusters of samples, each primarily associated with one diagnosis group (Fig. 3). Inter-patient variability significantly exceeded intra-patient variability (unpaired two-tailed *t*-test, *P* < 10^−4^), e.g*.*, with mean Bray–Curtis similarity in peri-implant mucositis of 51% between sites within the same patient versus 30% between different patients. In our study, we identified 228 of the 325 unnamed provisional species from the oral cavity (33), assigned with Human Microbiome Taxon (HMT) numbers. Currently, 64% of unnamed eHOMD species remain uncultured. Of the 228 species identified here, 157 met the relative abundance threshold of 0.1% across all samples (Table S5). These species corresponded to a mean of 9.3% of the analyzed communities (maximum 36%), with *Fusobacterium* sp. HMT-203 being the most abundant. Unnamed species with HMT numbers were more abundant in PIM (10.6 ± 7.7, mean ± SD, Kruskal–Wallis *P* = 0.013) and PI (12.1 ± 6.8, mean ± SD, Kruskal–Wallis *P* = 0.0004) compared to PIH (7.0 ± 5.9). Currently, the unnamed eHOMD species make up 35.4% of oral taxa, and 64% of them are yet to be cultured.

In summary, microbial communities on implants exhibited remarkable complexity, comprising a substantial proportion of understudied, yet unnamed, species. Notably, overall microbial diversity and abundance of anaerobic taxa increased in PI. At the genus level, PI was characterized by increased relative abundances of anaerobic genera, including *Prevotella* and *Dialister*, concomitant with reduced abundances of *Streptococcus* and *Rothia*.

### Peri-implantitis is characterized by high bacterial RNA yields and complex biofilm morphology

To capture microbial community functions beyond the microbiota composition detailed above, we incorporated a metatranscriptomic perspective in our investigation. The amount of bacterial RNA isolated from peri-implant samples and reflecting total transcriptional biofilm output was determined by multiplying the total RNA by the proportion of reads mapped to bacteria. The results were validated by the rRNA signals observed in Bioanalyzer profiles of the total RNA. The total amount of bacterial RNA varied significantly across diagnosis groups (Kruskal–Wallis, *H* = 24.5, *P* = 4.7 × 10^−6^; Fig. S1a). The median bacterial RNA amount increased from 1 ng in PIH to 10 ng in PIM (*post hoc* Dunn’s test, Bonferroni-adjusted *P* = 0.0096), and 50 ng in PI (*P* = 2.1 × 10^−6^ and *P* = 0.04 compared to PIH and PIM, respectively). A striking difference of four orders of magnitude was observed in bacterial RNA levels across samples, ranging from 0.05 to 1,500 ng. Higher total bacterial RNA is interpreted as indicating increased overall transcriptional activity and/or biomass of the biofilm, whereas lower RNA levels indicate reduced transcriptional output and/or biomass.

Notably, samples with exceptionally high RNA yields corresponded to generally high amounts of plaque quantified macroscopically using plaque index during sampling and processing (Spearman ρ = 0.49; *P* = 1.75 × 10^−8^; 95% CI 0.34–0.62, Fig. S1b). Median RNA yield increased from 2.51 ng at plaque index = 0–54.62 ng at plaque index = 3, and RNA yield differed across plaque index values (Kruskal–Wallis *P* < 0.0001). The association was not driven by outliers (ρ = 0.52 after removing the highest yield sample) and remained significant after adjusting for diagnosis in a log10 linear model (plaque index term *P* = 0.020).

Electron microscopy was performed on four PI samples with extensive biofilm colonization to characterize their morphology (Fig. 1b; Fig. S2). These biofilms displayed complex structures, which included cell filaments, bacterial aggregates, extracellular networks, and localized granules of extracellular polymeric substances (EPS), as well as features involved in biofilm stability, communication, and dispersal/remodeling, such as vesicles and motile flagellated cells.

### Metatranscriptomics revealed four major active CTs in peri-implant biofilms

**Fig 4:**
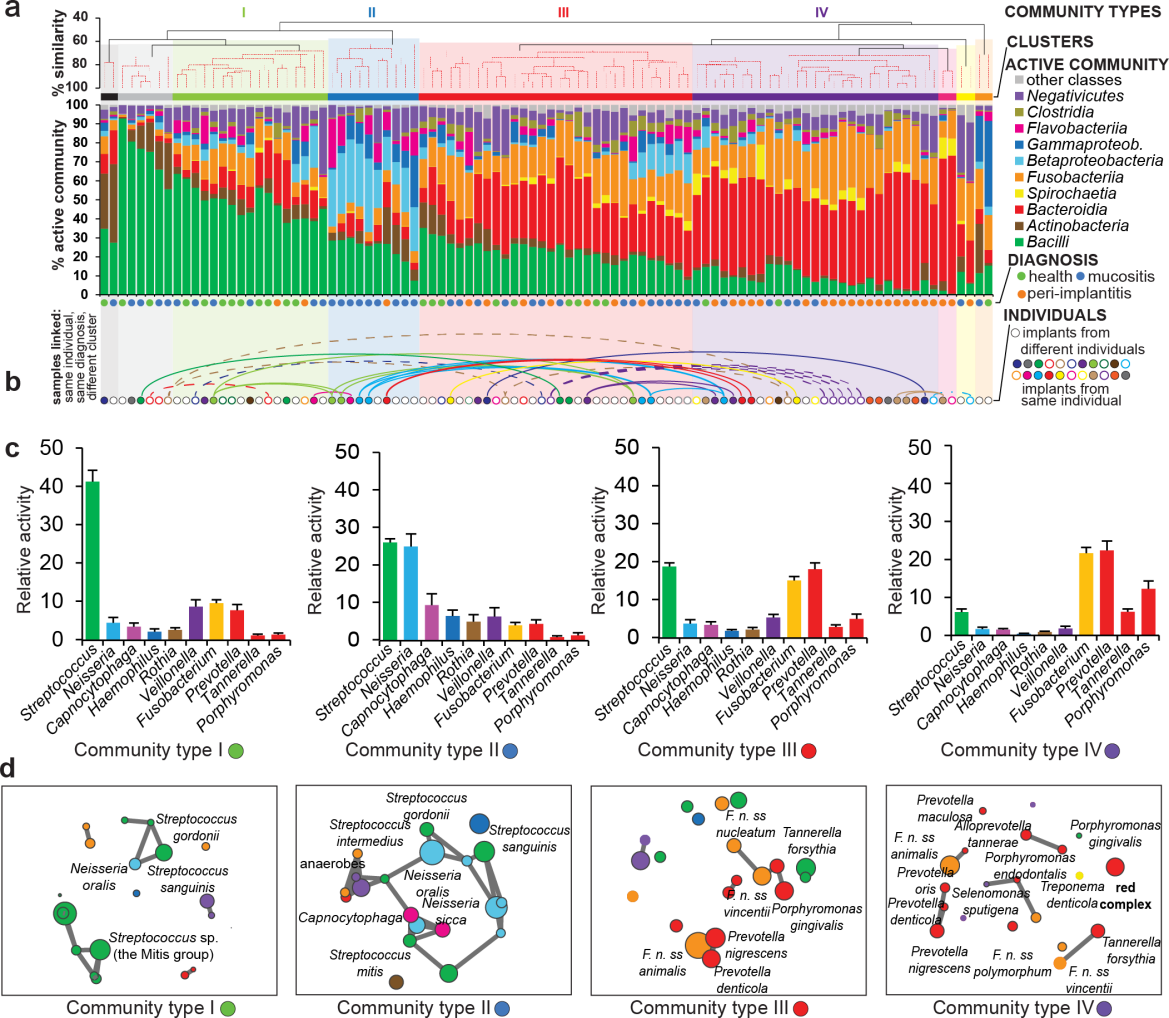
Major active community types at dental implants. (**a**) Clusters of class-level activity identified by SIMPROF. The main clusters representing major active community types (CT) were numbered I to IV and color-coded. The composition of the active community was plotted for each sample. Diagnosis is given below. (**b**) Inter-patient diversity. Samples originating from the same individual are highlighted and linked by lines if they are placed in different clusters despite the same diagnosis. (**c**) Most active genera. (**d**) Species correlation networks.

Metatranscriptomics allowed us to capture significant differences in activity distribution by analyzing the overall mRNA abundances across bacterial classes (Fig. 4; Fig. S3). It generated a total of 2.6 billion reads across 98 samples, with an average of 26 million reads per sample. Based on their activity profiles, biofilms at the class level showed statistically significant clustering (type 1 SIMPROF test, significance level of 0.1%). Importantly, clustering was not based on arbitrary similarity thresholds but was determined using the SIMPROF (similarity profile) algorithm, which employs permutation-based testing to identify statistically significant groupings within hierarchical clustering, thereby offering a robust approach to identify genuine clusters. Nine clusters of active communities, with four major clusters (CTs I–IV; Fig. 4a) covering most samples, were identified. Each CT displayed a unique profile of active bacterial classes. CT I was primarily associated with PIH samples and negatively associated with clinical measures reflecting disease severity. CT II almost exclusively included PIM samples, while CT III exhibited PIM as the highest proportion. CT IV had the highest proportion of PI samples. Across all cases, an average of ~30% of samples clustered together within the same SIMPROF group (Fig. 4b). In detail, complete lack of clustering was frequently observed. CT I was characterized by high Bacilli activity mainly from *Streptococcus*Fig. 4c). CT II was dominated by Betaproteobacteria activity, particularly *Neisseria*. Amplicon sequence variant analysis confirms that the detected taxa correspond to typical oral *Neisseria* species and not to classical pathogens such as *N. meningitidis* or *N. gonorrhoeae* (Fig. S4). Notably, *Neisseria oralis* expanded in peri-implant mucositis. CT III showed elevated activity of Bacteroidia (*Prevotella*) and Fusobacteriia (*Fusobacterium*), while CT IV exhibited even higher Bacteroidia activity, specifically from *Tannerella* and *Porphyromonas*, in contrast to CT III, along with increased Fusobacteria activity. The species co-occurrence network visualized through ordination showed unique networks of pairwise correlations between active species within CTs. CT II displayed the highest number of co-occurring taxa (Fig. 4d). Hence, clustering of active biofilm profiles identified four major community types, with CT II and CT III being predominantly associated with PIM.

Across diagnostic groups, DNA- and RNA-derived abundances were strongly correlated at the class level (Spearman *r* = 0.61–0.95; mean *r* = 0.81 ± 0.09 SD), indicating that transcriptional output largely paralleled community composition (Fig. S5a). Saccharibacteria represented a notable outlier (*r* = 0.30), whereas Deltaproteobacteria, Anaerolineae, SR1, and Clostridia showed comparatively weaker concordance (*r* < 0.75). Following DNA-based normalization (Fig. S5b, Table S6), only a restricted subset of classes, most prominently Fusobacteriia, Bacteroidia, Flavobacteriia, and Gammaproteobacteria, exhibited enriched RNA abundances with moderate effects (mean log₂ fold-change 0.54–0.66), including a pronounced enrichment of Fusobacteriia in health and Gammaproteobacteria in peri-implant mucositis (mean log₂ fold change 1.00 and 0.87, respectively).

### Microbial biofilm changes in peri-implantitis trigger numerous catabolic and a few specific anabolic pathways

**Fig 5:**
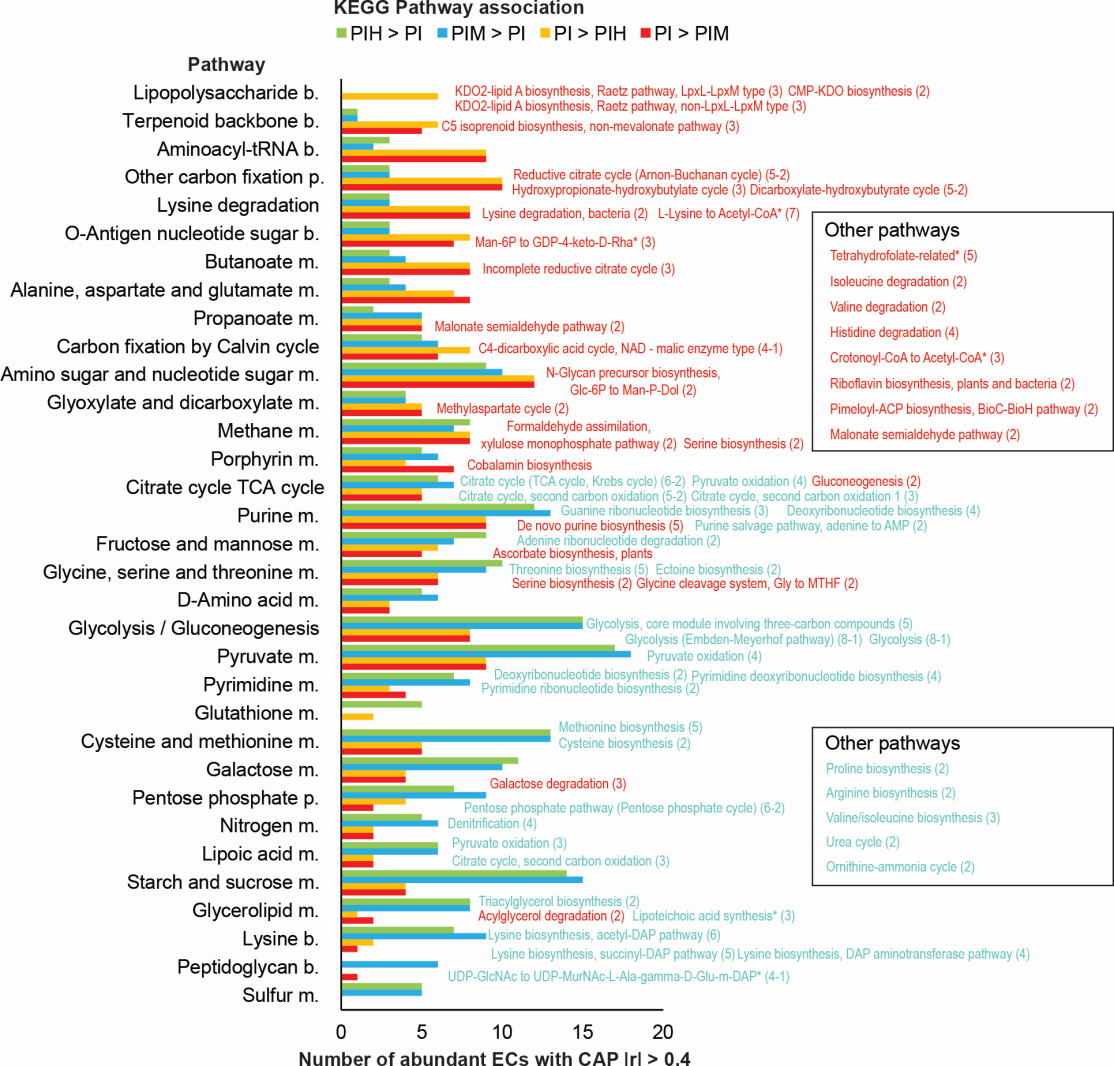
Pathways and modules associated with diagnosis groups. For each diagnosis group, the bar plot displays the number of enzymes (ECs) from specific KEGG maps that were correlated with the corresponding diagnosis using CAP. Individual KEGG modules are listed beside KEGG map bars if at least two members were associated with either PIH, PIM, or both (in cyan), or with PI in either or both comparisons (in red). PIH-PI and PIM-PI yielded largely overlapping results for both positively and negatively associated features with PI. In contrast, PIH-PIM showed no significant associations. Modules containing both PI-associated and non-PI-associated enzymes were excluded unless they were clearly dominated by one group (with a difference of at least three member ECs). In the brackets following each module name, the number of associated members is shown, if applicable, minus the count from the opposite group. Consecutive reactions spanning three or more steps not assigned to a KEGG module but showing correlation are labeled as custom reactions, indicated by an asterisk.

Next, we analyzed changes in transcriptome-level enzyme expression, categorizing them into Enzyme Commission (EC) numbers, which provide a numerical classification for enzyme-catalyzed reactions. Taxon-independent analyses offer the advantage of capturing microbial functions rather than focusing on specific taxa. Given the strong patient specificity of peri-implant biofilms and the functional redundancy among microbes, this approach enables detection of consistent, cross-patient signals that may be missed by taxon-focused methods. RNA-seq identified transcripts associated with 1,824 ECs, of which 36% had a relative abundance of at least 0.1% in at least one sample. The overall expression patterns in PI were distinct from both PIH and PIM (PERMANOVA *P* = 0.0001), whereas PIH and PIM comparison did not show significant differences in their EC-based profiles (*P* = 0.488) (Fig. S6). Using CAP and DESeq2 analyses, we identified ECs with significantly elevated expression in individual diagnosis groups (Table S7). We then mapped these diagnosis-group-associated ECs onto KEGG pathways to assess their associations within specific KEGG modules or sequential reaction chains (Fig. 5). ECs positively associated with PIH or PIM were—in comparison to PI—primarily linked to anabolic processes, including the synthesis of multiple amino acids, glycerolipids, pyrimidines, and purines, as well as to pathways involved in denitrification, the pentose phosphate cycle, glycolysis, and the citrate cycle. ECs positively associated with PI represented catabolic processes, such as degradation of multiple amino acids, acyloglycerols, and galactose. Additionally, some ECs involved in biosynthetic pathways, such as those for isoprenoids, purines, and serine, as well as carbon fixation and gluconeogenesis, were also positively associated with PI. Among the top CAP-associated ECs distinguishing PIH and PI, PIH showed a lower catabolism-to-anabolism ratio based on aggregated relative read abundances compared with PI (linear mixed-effects model, *P* = 0.0057). A similar pattern was observed based on EC category counts, although this was not statistically significant (Fisher’s exact test, *P* = 0.128). This pattern was mainly driven by a higher aggregate abundance of anabolic marker ECs in PIH, whereas differences in the numbers of anabolic and catabolic marker ECs played a secondary role.

In addition to the diagnosis groups, ECs were also analyzed across the RNA-seq activity-based CTs. PIM samples representing CT II and CT III differed in the expression of 219 ECs (*P* < 0.05; Table S8). PIM CT II was mainly linked to pyruvate and lipoic acid metabolism, while CT III was characterized by lysine degradation and butanoate metabolism, which are functionally interconnected via shared CoA intermediates and butyrate production (crotonyl-CoA/butyryl-CoA branch). Finally, changes in amino sugar and nucleotide sugar metabolism were observed in both PIM-associated CTs. These data, along with biofilm biomass estimates derived from bacterial RNA yields, indicate that the difference between PIH and PIM is predominantly driven by biofilm expansion, accompanied by only limited changes in community composition and functional activity. In contrast, the PIM and PI differences involve both biofilm expansion and significant functional alterations.

### Key microbial players demonstrate class-specific functions

**Fig 6:**
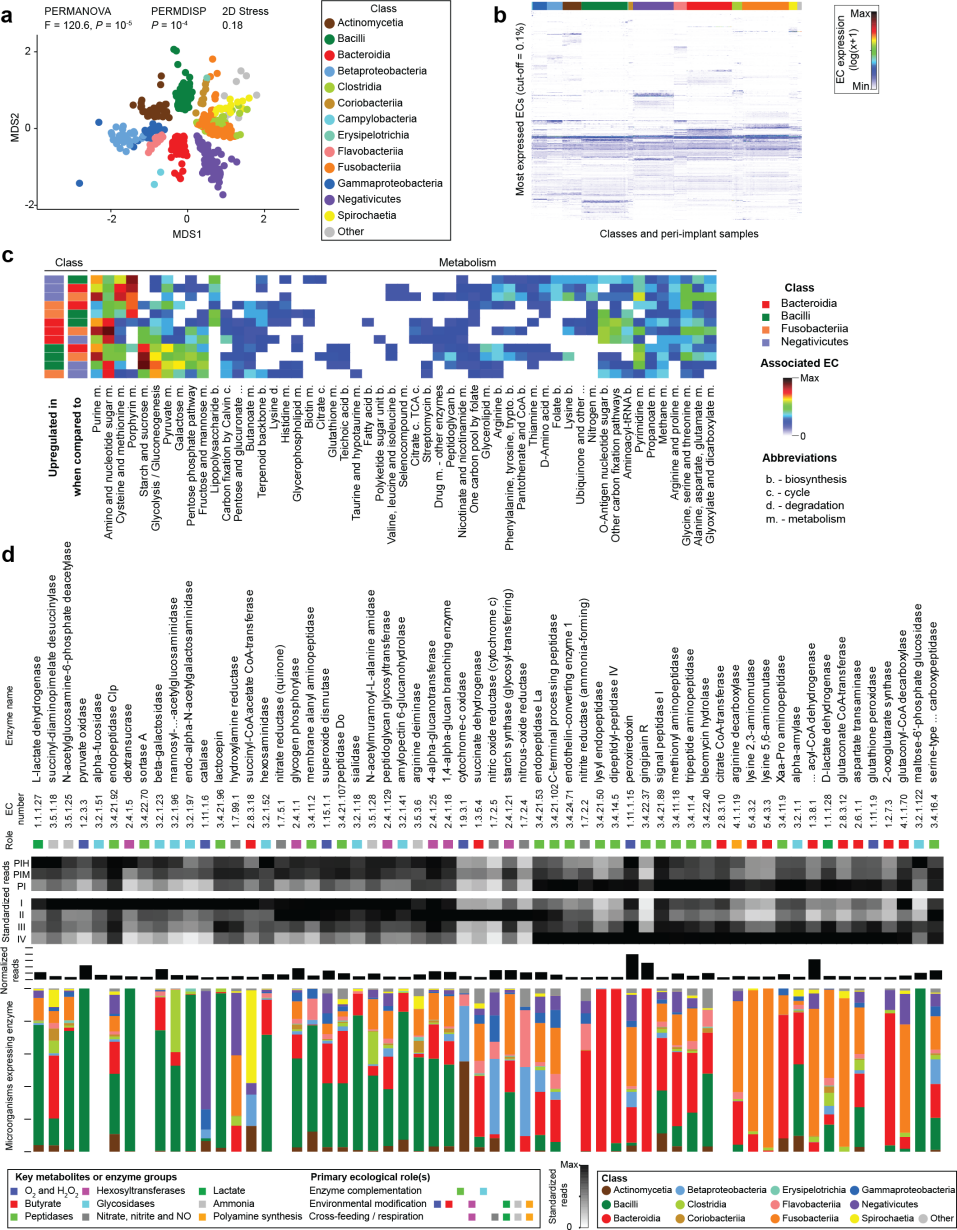
Unique functions of microbial classes in peri-implant biofilms. (**a**) Unconstrained ordination of EC-level expression data, with the microbial classes in each sample treated as a separate sample, resulting in *n* = 541 pseudo-samples. MDS plot calculated on Bray–Curtis dissimilarities for log(*x* + 1)-transformed read counts grouped to enzymes (E.C., relative activity cutoff = 0.1%, number of ECs = 1,370). Data points are colored by class. (**b**) Shade graph for class activity across samples. Class-sample combinations and enzymes were each sorted using hierarchical clustering on Euclidean distances. Same input data as for panel **a**. (**c**) Shade graph for class-specific activities. ECs whose activity was associated with a given class were grouped into KEGG maps. “max” refers to the maximum relative abundance (i.e., transcriptional activity) observed for a given EC across samples. (**d**) The top 60 most highly expressed ECs with predicted ecological relevance were analyzed. ECs were grouped based on Spearman correlations, and their expression levels were standardized by their maximum values and visualized as a heatmap across diagnostic groups and community types. Below, normalized counts and the composition of producers are illustrated. ECs are color-coded by ecological groups, defined by their associated metabolites, enzymes, and ecological roles. Producer data are presented at the class level. The empty bars represent ECs that could not be confidently assigned to a specific microbial taxon by our annotation pipeline. These ECs are retained in the taxon-independent EC abundance table but are excluded from the taxon-stratified EC-class analysis.

To understand the physiology and functional roles of microbial members within the biofilm, we examined class-specific activities in peri-implant biofilms using taxon-standardized transcriptional profiles (Fig. 6; Table S9), thereby complementing the community-wide DNA and RNA profiles presented in the previous sections. Ordination (Fig. 6a) and clustering (Fig. 6b) revealed unique activity fingerprints for each class (Fig. 6b). Diagnosis-related differences become more apparent when individual taxa are analyzed separately, e.g*.*, Negativicutes (PERMANOVA pseudo-*F* = 6.9, *P* = 0.0001). Pairwise comparisons of the four most active bacterial classes (*P* < 0.05) identified class-associated ECs that were subsequently grouped into KEGG maps (Fig. 6c). Bacilli, associated with PIH/PIM (often representing CT I), exhibited higher activity in carbohydrate, nucleobase, and pyruvate metabolism, as well as the pentose phosphate pathway. Negativicutes, also PIH/PIM-associated, showed enriched nucleobase, porphyrin, lipopolysaccharide, and amino acid metabolism. Bacteroidia, PI-associated as well as abundant and highly active in CT III/CT IV, were characterized by amino sugar, purine, and one-carbon metabolism. Fusobacteria exhibited porphyrin metabolism, similar to Negativicutes.

To uncover the ecological functions of peri-implant microbial classes, we linked the ECs to their ecological roles and associated metabolites, analyzing the top 60 most highly expressed features across diagnosis groups and CTs (Fig. 6d; Table S10). Glycosidases from Bacilli and peptidases from Bacteroidia, known to participate in enzyme sharing for synergistic degradation of host-derived polymers like salivary glycoproteins (34, 35), showed the highest expression in PIH/PIM and PI, respectively, with β-galactosidase (EC 3.2.1.23) and Arg-gingipain (EC 3.4.22.37) being the most highly expressed enzymes. H_2_O_2_ production (EC 1.2.3.3), primarily driven by Bacilli and Negativicutes-driven H_2_O_2_ detoxification (EC 1.11.1.6), was observed in PIH and PIM. Among oxidative stress-related enzymes, peroxidases (EC 1.11.1.15) showed the overall highest expression. Hexosyltransferase activities from Bacilli were indicative of carbohydrate polymer biosynthesis, while butyrate and polyamine production driven by Fusobacteria was associated with PI. Notably, the PIM CT II subtype exhibited unique activities, including oxygen depletion (EC 1.9.3.1), succinate/fumarate metabolism (EC 1.3.5.4), denitrification (EC 1.7.2.4/5), and glycogen synthesis (EC 2.4.1.21), highlighting distinct ecological adaptations in peri-implant biofilms.

### Bacteriophage activity clusters with different types of peri-implant disease

**Fig 7:**
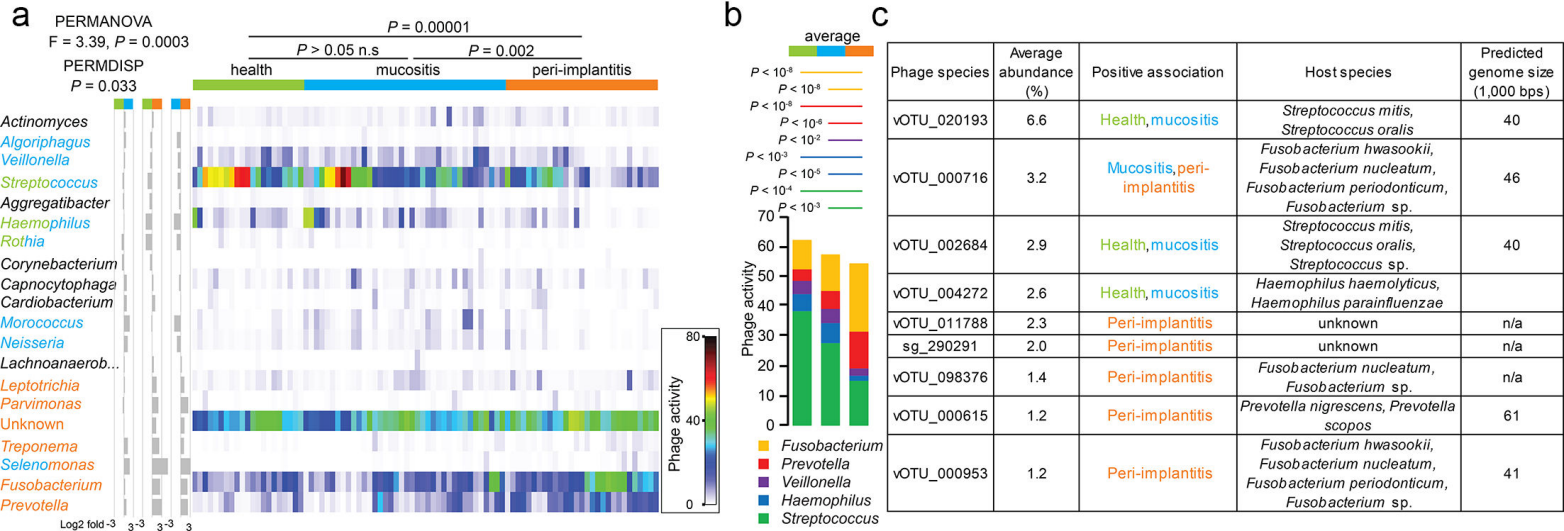
Active phageome across diagnosis groups. (**a**) Heatmap for phages grouped by a host. Diagnosis associations are indicated by font color. (**b**) The activity of phages infecting five genera dominated the biofilms and exhibited high diagnosis specificity. (**c**) Most abundant peri-implant phage species (average relative activity cutoff = 1%). Green, blue, and orange colors represent peri-implant samples from healthy, mucositis, and peri-implantitis, respectively. All listed species were predicted to exhibit a temperate lifestyle, with an average Phage TYPe prediction tool (PhaTYP) score of 0.93. IMG/VR host assignments are presented and were concordant with CHERRY-based genus-level predictions in all cases (*n* = 7).

Despite their critical role in shaping bacterial populations and influencing biofilm physiology, the peri-implant phageome remains poorly characterized (36). To replicate, oral bacteriophages (predominantly DNA viruses) must hijack the host bacterial machinery. As a result, MTX captures transcripts derived from active phages and phage-like elements, providing a functional readout of phageome activity. Using the IMG/VR database as a reference, we assessed transcription levels of phage-like genes, grouped as provisional species-like vOTUs (i.e., viral operational taxonomic units representing sequence bins). Ordinations based on phage data showed distinct separation between PIH, PIM, and PI samples (PERMANOVA *P* = 6 × 10^−5^). CAP and DESeq2 analysis revealed phage species (vOTUs) associated with each diagnosis group (Fig. S7; Table S11). Analysis of phage hosts revealed high activity of phages targeting typical oral microorganisms with distinct patterns across diagnosis groups. However, a significant fraction of phage activity remained unassigned to specific bacterial hosts (Fig. 7a). Phages targeting *Fusobacterium* and *Prevotella* exhibited higher activity in PI than in PIH or PIM, where phages targeting *Streptococcus* and *Haemophilus* were more active (Fig. 7b). Additionally, *Veillonella* phages showed greater activity in PIM than in PI. The *Streptococcus* phage, vOTU 20193, exhibited the overall highest average relative activity of 6.6% across all samples (Fig. 7c). All of the most active phage species were classified as temperate. Thus, transcripts associated with the most transcriptionally active phage-like elements likely reflect gene expression by temperate DNA phages during lysogeny or prophage induction. In conclusion, variations in the active phageome across diagnosis groups reflected changes in both the abundance and activity levels of phage hosts.

### Peri-implant disease exhibits a distinct host response to biofilm

**Fig 8:**
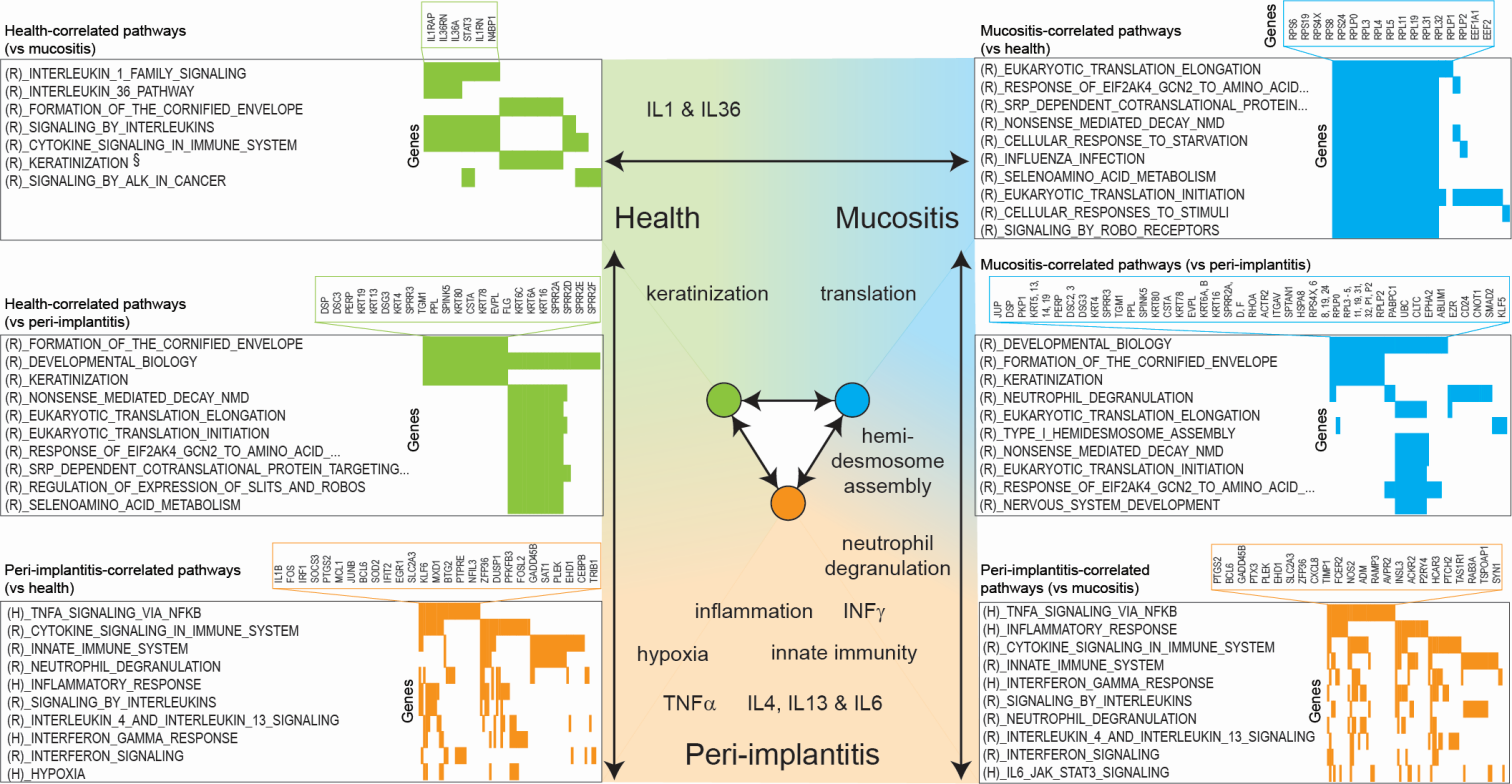
Host gene expression across diagnosis groups. Pathways representing genes correlated with diagnosis groups (ǀ *r* ǀ > 0.25) in CAP. All or top 10 pathways are listed for each comparison and diagnosis group based on increasing adjusted *P* value *q*-value. Genes representing the top pathway are listed. (Ranges of adjusted *P*-values from top pathway [from top left graph, clockwise]: 5.5 × 10^−5^–1.1 × 10^−2^, 3.2 × 10^−29^–1.0 × 10^−21^, 1.7 × 10^−30^–1.0 × 10^−14^, 3.2 × 10^−40^–5.1 × 10^−10^, 1.2 × 10^−32^–6.0 × 10^−10^, 2.0 × 10^−25^–1.5 × 10^−14^.) (H), hallmark gene sets; (R), reactome subset of canonical pathways. Pathways enriched in PIH are shown in green, those enriched in PIM are in blue, and those enriched in PI are in orange.

The peri-implant sulcus serves as a unique ecological environment where microbial biofilms interact with peri-implant crevicular fluid. Detection of intact human 18S and 28S ribosomal RNA species, indicating higher transcript integrity, enabled minimally invasive transcript-level analysis of host activity at the local host–microbiome interface. Detected host-derived RNA originates from shed host cells and extracellular RNA present within the biofilm matrix. We examined the expression of approximately 35,000 human genes (excluding low-count genes) around dental implants across diagnosis groups using DESeq2 and CAP analysis (Fig. S8). The host gene expression patterns in PI differed significantly from both PIH (PERMANOVA *P* = 0.011) and PIM (PERMANOVA *P* = 0.004). In contrast, the comparison between PIH and PIM showed no significant differences in their profiles (*P* = 0.33). DESeq2 analysis revealed 102 differentially regulated genes when comparing PI and PIM, with 32 genes upregulated and 70 downregulated in PI (DESeq2 adj *P* < 0.05), and the smallest number of differentially regulated genes in the PIM vs PIH comparison (three genes upregulated in PIM). Using CAP analysis, we identified the top actively expressed genes (filtered at a 0.1% maximum cutoff) which showed a high correlation (ǀ *r* ǀ > 0.25) with diagnosis groups. We further identified enriched GSEA hallmark gene sets, which represent specific well-defined biological processes and canonical pathways, associated with diagnosis groups (adjusted *P* < 0.05, tested for CAP outputs; Fig. 8). This analysis showed that keratinization was linked to PIH, while increased expression of genes relating to translational activity was characteristic of PIM compared to PIH. PIM samples representing CT II and CT III differed by the expression of only 12 host genes. Comparisons between PIH vs PI and PIM vs PI revealed similar host pathways associated with both PIH and PIM, primarily linked to keratinization and translation. An increased abundance of transcripts associated with hemidesmosome assembly was notably characteristic of PIM in the PIM vs PI comparison. PI signature pathways were largely consistent across comparisons, predominantly associated with TNF-alpha signaling via NF-κB, inflammatory responses, cytokine signaling, interferon signaling, and hypoxia. In summary, the host response in PIH and PIM was similar, reflecting an intact epithelial barrier and housekeeping functions, while PI was strongly associated with inflammation and related signaling.

### Multi-omic integrative analysis revealed complex interactions in the peri-implant ecosystem

**Fig 9:**
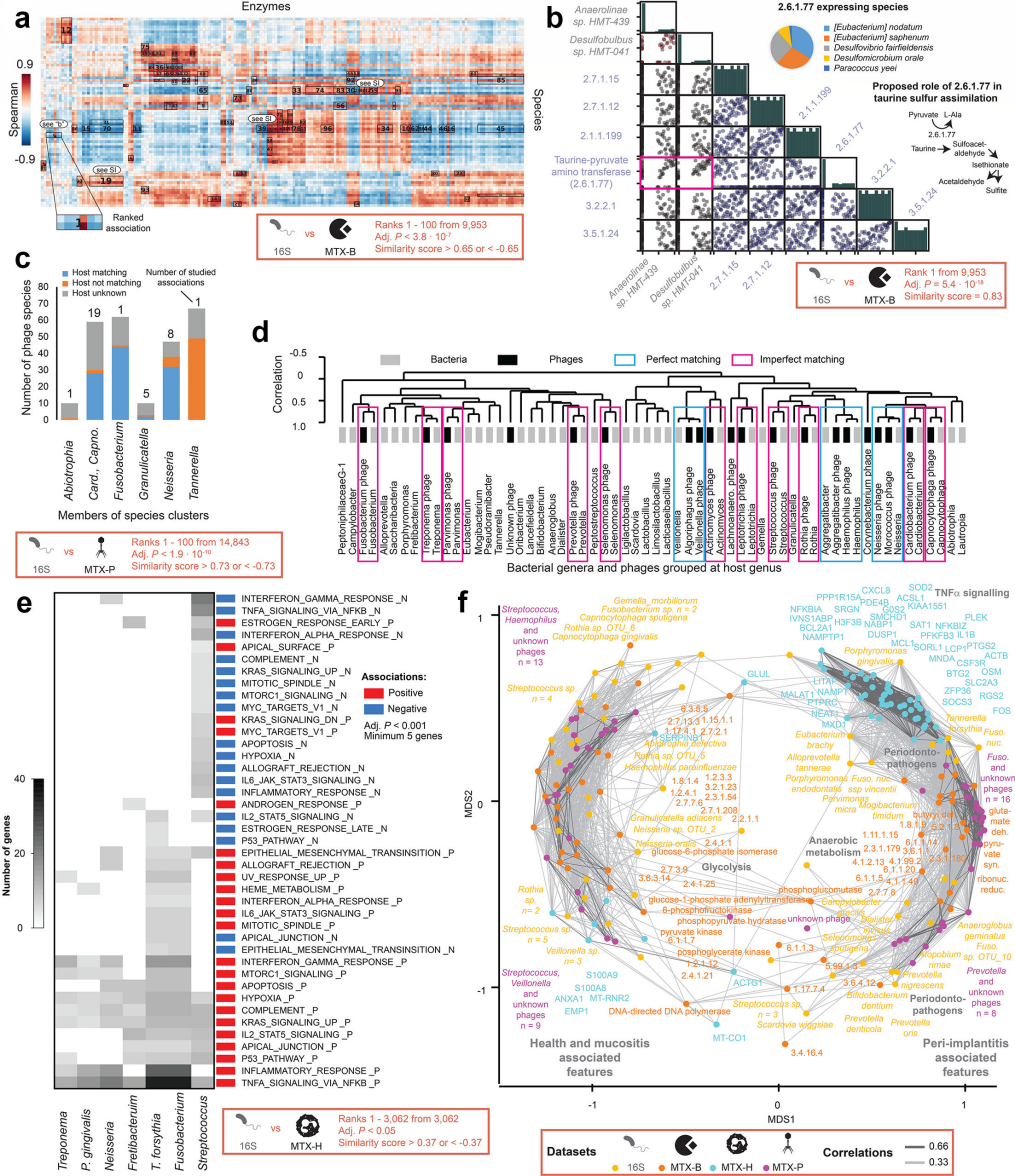
Network of interactions between microbial, ecological, biochemical, and host factors. (**a**) Associations between species clusters (at DNA level) and enzymes (at RNA level), with the top 100 associations displayed on the heatmap. (**b**) The strongest association from panel **a**. The highlighted species were not identified as producers of the corresponding enzyme. Instead, the predicted enzyme-producing taxa are summarized in the accompanying pie chart. (**c**) Relationship between phage-associated species and predicted hosts of those phages. (**d**) Correlations between genera and phages grouped by their host genus. (**e**) Relationship between species (at DNA level) and host response (at RNA level). (**f**) Integrative clustering analysis of four data sets.

We leveraged our multi-omics, multi-layered data set to trace key interactions within the peri-implant ecosystem by investigating pairwise interactions among bacterial species abundances (full-16S-based), enzymes, phages, and host genes (all MTX-based), using hierarchical association testing (Fig. 9). This was followed by integrative analyses combining features from all data sets.

Profiling of species–enzyme relationships revealed potential interspecies metabolic dependencies (Fig. 9a; Table S12). The strongest association, identified from approximately 10,000 statistically significant correlations, was a positive correlation between Anaerolineae sp. HMT-439, *Desulfobulbus* sp. HMT-041, and taurine–pyruvate aminotransferase (EC 2.6.1.77), an enzyme involved in taurine sulfur assimilation, predominantly expressed by *Eubacterium*, *Desulfovibrio,* and *Desulfomicrobium* species (Fig. 9b). Thus, our integrative analysis identified distinct clusters of highly correlated species and enzymes, further distinguishing between active species that directly express these enzymes as shown by the EC transcripts assigned to them, and other species that, despite lacking expression capabilities, are likely to be associated with or dependent on these activities.

The species-level analysis identified 14,843 significant bacterium-phage associations (Table S13). In most cases, clusters of phage species/vOTUs were associated with clusters of their predicted bacterial hosts. An exception was observed for phages predicted to infect *Fusobacterium*, which also showed associations with *Tannerella forsythia* (Fig. 9c). The genus-level analysis demonstrated near-complete concordance between phages and their hosts (Fig. 9d).

The microbial taxa (full-16S) and host response genes (MTX) analysis identified 7,482 associations (Table S14). The top 10 associations comprised five clusters representing nine predominantly disease-associated microbial species and seven clusters encompassing 40 host genes (FDR-adjusted *q* < 7.06 × 10⁻⁷, Spearman’s ρ > 0.67). Unconstrained analysis was followed by an analysis of selected associations of representative key biofilm members (Fig. 9e). In all, 107 associations were found between representative taxa and host genes. They were grouped into 24 hallmark sets. *Streptococcus* exhibited a distinct profile characterized by a high number of negative associations. *Neisseria* grouped with disease-associated taxa, while *Fusobacterium* clustered closely with *T. forsythia*. Additionally, *P. gingivalis* displayed a profile similar to that of *Treponema*.

At the functional level, we identified 36,617 associations between bacterial enzymes (MTX) and host response genes (MTX) (Table S15). Gingipain R, regarded as one of the most important enzymes causally linked to periodontal and peri-implant pathologies (37), in cluster with mannanase (3.2.1.25), was positively associated with 61 host gene clusters representing TNFα signaling (FDR-adjusted *q* = 6.5 × 10^−35^), inflammatory response, and 12 other hallmark gene sets.

Integrative clustering analysis of the most abundant and active elements across four datasets consistently depicted the peri-implant ecosystem (Fig. 9f). The major distinctions between PIH/PIM and PI were evident at all molecular and taxonomic levels.

## DISCUSSION

In this study, we utilized the largest clinical cohort to date with paired microbiome composition and metatranscriptomic profiling of implant-associated biofilms to integrate multiple molecular features and present the first high-resolution *in situ* characterization of the peri-implant ecosystem across PIH, PIM, and PI. Consistent with our prior investigations, inter-patient variability exceeded intra-patient variability, indicating that peri-implant biofilm composition and activity, although condition dependent, are highly subject specific (31, 38). While overall population dynamics aligned with patterns observed in previous studies on microbiome composition (7–9, 12, 14, 39–42), our integrative approach, incorporating metatranscriptomics, revealed novel disease-associated features. These findings include total transcriptional biofilm output, substantial site-specific functional heterogeneity, active community types, the activity of ecologically relevant enzymes and phages, as well as characteristic host response patterns. Furthermore, association testing provided a broader context by uncovering potential bacterium–enzyme, phage–bacterium, and microbe–host interactions across different molecular levels. These insights provide a deeper understanding of the physiology, ecology, and virulence of peri-implant biofilms; however, the cross-sectional design precludes inference of temporal relationships or causality and warrants cautious interpretation.

Both clinical and molecular measurements, including total transcriptional biofilm output, consistently showed that biofilm expansion is a primary driver of the PIM development around implants and contributes to its further progression to PI. Our findings revealed highly conserved enzyme expression among biofilm members, pointing to substantial functional specialization and interdependence. Combined with the high species diversity, these characteristics may promote strong growth synergies within the biofilm (43). Additionally, observed variation in host response, including non-canonical response profiles within diagnostic groups, suggest an important role in biofilm regulation (44, 45).

At the RNA level, biofilm-standardized transcriptional activity, abundance-normalized biofilm activity, and taxon-normalized activity captured complementary dimensions of functional contribution by individual biofilm members. Together, these metrics delineated diagnosis-associated shifts, identified taxa exhibiting elevated transcriptional output relative to genomic abundance, and resolved within-taxon physiological adaptations. Consistent with prior oral microbiome studies (31, 46–50), DNA and RNA abundances were strongly correlated across taxa and diagnostic groups, indicating that transcriptional output broadly tracks community composition. Notably, Saccharibacteria, Deltaproteobacteria, Anaerolineae, SR1, and Clostridia exhibited comparatively weak DNA-RNA concordance (*r* < 0.75), consistent with the limited genomic resolution of several hard-to-isolate and under-characterized oral taxa within these lineages (51). After DNA-based normalization, Fusobacteriia and Gammaproteobacteria emerged as the most transcriptionally enriched classes in peri-implant health and mucositis, respectively. The role of Fusobacteriia as ecological hubs within oral biofilms is well established (52), whereas the prominence of Gammaproteobacteria highlights a potentially underappreciated, diagnosis-associated functional contribution. Given that peri-implant microbiology has historically emphasized obligate anaerobes, the ecological roles of Gammaproteobacteria and their phages warrant further targeted investigation (53–55).

The potential role of oral *Neisseria,* generally regarded as low-virulent (56), in PIM merits focused discussion, as consistent signals emerge across both DNA- and RNA-based data sets. Based on full-16S, *Neisseria*, particularly *Neisseria oralis*, was significantly enriched in PIM. MTX further identified an alternative PIM-associated community type characterized by high *Neisseria* activity. Notably, our integrative full-16S-MTX correlation analysis indicates that these taxa may be associated with host-response patterns resembling those induced by established periodontopathogens. The literature presents a heterogeneous picture: some DNA-based clinical studies associate *Neisseria* with PIH (11, 14, 40, 41); others link it to PIM (6) or less severe PI (42), or report no association but generally high abundance (57). This variability suggests a context-dependent or potentially dual role in oral health and disease. Recent evidence further implicates commensal *Neisseria* as an early contributor to peri-implant dysbiosis, particularly in individuals with a history of periodontitis (58); similar niche-dependent pathogenic behavior has been described in airway diseases (59, 60). Although oral *Neisseria* encode multiple established and putative virulence factors, their roles in peri-implant infections remain largely uncharacterized (61). In our data set, the specific association of *N. oralis* with PIM suggests that the inflamed peri-implant niche may select for particular *Neisseria* taxa. At the same time, limitations of partial 16S rRNA gene-based classification complicate comparisons across studies and underscore the need for improved methods to quantify and differentiate oral *Neisseria* taxa (62–64).

Several factors may regulate *Neisseria* populations. Bleeding, a hallmark of PIM, provides heme, potentially supporting *Neisseria* dominance (65). However, under anaerobic conditions, *Neisseria*’s activity diminishes unless alternative electron acceptors, such as nitrate, are available, potentially sourced from diet or biofilm metabolism (60, 66, 67). This dynamic could explain the presence of CTs with or without high *Neisseria* activity. Our findings suggest that *Neisseria* may contribute to the early peri-implant pathology in patient subpopulations. Further investigation into host*–Neisseria* interactions in the peri-implant environment is warranted to better understand their potential role as a pathobiont. Additionally, experimental (68) or observational (69) longitudinal clinical studies could provide further insights into whether *Neisseria* activity modulates the progression dynamics of mucositis.

For the first time, we specifically analyzed the expression of enzymes that were previously reported or predicted to be ecologically relevant (34), aiming to assess their functional significance in the peri-implant ecosystem. As expected, the most highly expressed ECs with predicted ecological function were involved in oxidative stress generation or reduction, enzyme sharing, and metabolic cooperation. While we confirmed several important contributors, e.g*.*, enzymatic complementation involving glycosidases and proteases, trophic chains linked to short-chain fatty acid production, and environmental modification through oxygen utilization and ROS detoxification (70–74), we also identified ecologically active enzymes and taxa that have not yet been included in existing interaction models and warrant further investigation, e.g*.*, components of the denitrification network.

Community type- or disease state-specific patterns revealed potential target interactions for anti-biofilm interventions. Beyond bacterial activity, the peri-implant phageome emerges as a significant, yet understudied (36) component of peri-implant biofilms. In the present study, we uncovered a remarkable diversity of active oral phages and phage-like elements within the peri-implant ecosystem, consistently identifying them as key biofilm constituents across diagnostic groups and microbial communities. However, the metatranscriptomic detection of phage-derived transcripts does not, by itself, demonstrate active lytic replication or bacterial lysis. Addressing this limitation will require linking *in vivo* transcriptomic patterns with *in vitro* systems capable of resolving early and late-lytic gene modules, including structural gene expression. Such efforts remain technically challenging, as isolation and cultivation of oral phages are often difficult (36). Synthetic communities co-cultured with human cells may provide an experimental framework to address this limitation, at least for selected phage phylotypes (75). The strong association between *Tannerella forsythia* and *Fusobacterium* phages suggests that phage-induced lysis may facilitate the expansion of neighboring fastidious species. These findings are supported by previous experiments showing that conditioned media from *Fusobacterium* spp. cultures promote the growth of fastidious microorganisms, including *Prevotella* spp., phylogenetically related to *Tannerella* spp. (76). Additionally, the activity of phages with unknown hosts correlated to the presence of specific bacterial species, thus uncovering potential hosts such as *Capnocytophaga* and *Fusobacterium* spp. Our approach could be applied to refine current host assignment methods for phages in the future.

Although plaque-derived host RNA may originate from exfoliated or lysed epithelial and immune cells, it reflects the host–microbiome interface *in situ* and provides valuable, minimally invasive insight into localized immune responses. This approach has been increasingly recognized in respiratory microbiome studies (23, 24), and exploratory analyses of host transcripts from oral samples such as saliva, buccal swabs, and gingival crevicular fluid have shown promise (77). The comprehensive profiling of the host response across disease states is associated with distinct host activity patterns in each diagnostic group. Notably, upregulation of keratinization in PIH (vs PIM) and in PIM (vs PI) emphasizes the critical role of keratinized epithelium in maintaining peri-implant health. Keratinized epithelium protects mucosal tissues from microbial insults, reducing vulnerability and tissue damage (78). Insufficient keratinized mucosa around dental implants has been linked to increased plaque accumulation, tissue inflammation, mucosal recession, and attachment loss (3, 79). These findings suggest that the stability of the soft tissues surrounding osseointegrated dental implants significantly impacts bone regeneration processes and long-term outcomes. PI was strongly associated with inflammatory responses, such as TNF-α signaling via NF-κB, cytokine signaling, interferon signaling, and hypoxia. TNF-α is a key mediator of periodontal inflammation and alveolar bone resorption (80, 81). The detection of host gene expression related to hypoxia in PI corresponds with the observed dominance of anaerobes in biofilms, which are major producers of lipopolysaccharides (LPS) (82). The hypoxic environment may enhance TNF-α and other cytokine expressions in response to biofilm-derived LPS, subsequently activating the NF-κB pathway (83). Together, these molecular signatures offer a coherent and detailed understanding of the pathological mechanisms underlying PI. Notably, host-derived signals obtained from a microbial-oriented metatranscriptomic workflow are inherently less controlled than those generated by dedicated host-targeted RNA-seq. Accordingly, these data should be interpreted with caution, as they may be influenced by variability in host cell content, RNA degradation, and capture efficiency. Validation against biopsy-derived transcriptomes will be important to establish biological relevance. Despite these limitations, dual RNA sequencing provides a valuable framework for integrated host–microbe analyses in peri-implant biofilms.

Our integrative analysis revealed an intriguing link between the PI-associated *Anaerolineae-Desulfobulbus* cluster and taurine–pyruvate aminotransferase, which is involved in anaerobic respiration in *Eubacterium*, *Desulfovibrio*, and *Desulfomicrobium*, as the strongest association. Taurine, a ubiquitous sulfonate metabolite, accumulates in inflammatory lesions (84, 85). *Anaerolineae* and sulfate-reducing bacterium “SRB” *Desulfobulbus* have been associated with periodontal disease (46, 50, 86–89), produce virulence factors (76, 90), and rely on scavenging essential metabolites due to the loss of some biosynthetic capabilities (90–92). This suggests that taurine-metabolizing *Eubacteriu*m spp. and two SRBs (*Desulfovibrio* and *Desulfomicrobium*) may provide growth factors or aid in the removal of inhibitory metabolites for the *Anaerolineae–Desulfobulbus* consortium. These fastidious and sensitive SRBs have also been linked to disease (93–96) and may depend on *Anaerolineae*, e.g*.*, for the breakdown of complex organic compounds or the production of growth factors, as similar interactions were observed in the gut for SRBs (97).

Although our study is the first to provide integrative insights into key microbial/molecular relations in peri-implant health and disease and potential drivers of dysbiosis by using multi-omics, certain limitations should also be considered, such as the lack of spatial information to measure ecological interactions and the associative or provisional nature of the findings. SEM analysis of macroscopically visible, large detached peri-implantitis biofilm fragments enabled qualitative assessment of major structural features but precluded reliable inference of their original orientation relative to the implant surface. Future studies using our *in vivo* abutment-based model for atraumatic biofilm retrieval, combined with confocal laser scanning microscopy and targeted probes, will enable spatially resolved identification of structural components, taxa, and transcripts (38, 98, 99). While our sample size is the largest in peri-implant metatranscriptomics to date, it does not establish causality. Longitudinal, biogeographical, and mechanistic studies are needed to better understand biofilm dynamics and site specificity, validate causal effects, and identify population-specific responses. Ongoing tissue and biofilm culture models developed in our group provide a controlled framework to mechanistically dissect strain-specific functions and host interactions prior to *in vivo* validation (100, 101). Application of culturomics will be critical to obtain representative, relevant but hard-to-culture bacterial and phage isolates for controlled *in vitro* models (51, 55). Especially the latter is scarce but highly needed to validate provisional phage species, improve the reference databases, as well as characterize their physiology and ecology (36). Nevertheless, our integrated multi-omics approach provides a robust framework for future ecological and clinical studies, with the ultimate goal of improving diagnostic, preventive, and treatment strategies for peri-implant disease.

## MATERIALS AND METHODS

### Study design and ethical statement

The present clinical cross-sectional study was conducted at the Department of Prosthetic Dentistry and Biomedical Materials Science, Hannover Medical School, Germany, as part of the “SIIRI Biofilm Implant Cohort (BIC).” SIIRI (Safety Integrated and Infection Reactive Implants) is an interdisciplinary initiative that aims to recruit hundreds of individuals and monitor their peri-implant microbiome over a period of up to 12 years to investigate the development of peri-implant diseases and devise early detection and prevention strategies (29). Clinical classifications for participants who had at least one dental implant were peri-implant health, peri-implant mucositis, or peri-implantitis, based on the 2017 World Workshop on the classification of periodontal and peri-implant diseases and conditions.Patient and implant characteristics are listed in Table S1. Detailed disease definitions and clinical examination, as well as inclusion and exclusion criteria, are described in Supplemental material.

### Sample collection and co-isolation of DNA and RNA

Peri-implant biofilm samples were collected from six sites per implant using sterile paper points (ISO 35/2.0, VDW GmbH) and curettes (HuFriedy Mfg. Co. LLC) to maximize input mass (102). Samples were pooled in RNAprotect (Qiagen), incubated for at least 5 minutes, and stored at −80°C until further processing. DNA and RNA were co-isolated using a published protocol (49, 103) with modifications. The sample collection and co-isolation procedures are described in Supplemental material.

### Full-16S rRNA gene amplicon sequencing and RNA sequencing

Full-length 16S rRNA gene amplicon sequencing (full-16S) was performed on a PacBio Sequel system, followed by bioinformatic analysis using an in-house pipeline (38). The data set included 125 samples with a total of 2,012,347 reads, with an average sequencing depth of 13,783 reads per sample.

For MTX analysis, ribosomal RNA was depleted using the Ribo-Zero Kit Epidemiology, and mRNA sequencing was performed on an Illumina HiSeq 2500 (50 or 68 base pairs). The quality-filtered sequences were mapped to a custom oral metagenome reference created from eHOMD (33, 104) and Pasolli et al. (105) and further annotated with eggNOG (106). To characterize functional features, counts were aggregated based on gene–EC relationships and taxonomic information (107). Phageome was characterized using the IMG/VR database as reference (108). Of 2.7 billion raw reads, 40% mapped to the human genome, and 1.4 billion were classified as bacterial or viral. In total, 350 million mRNA reads had EC annotation assigned.

The details of the full-16S and metatranscriptomic analyses are described in Supplemental material.

### Statistical analysis and association testing

Multivariate analyses were performed using PRIMER v7 with PERMANOVA+ (109, 110). Bray–Curtis dissimilarities or Euclidean distances of untransformed or transformed relative abundances were used for hierarchical clustering (group-average linkage, when applicable, combined with SIMPROF permutation tests) and ordinations (nMDS and PCoA). Variables were compared across groups using PERMANOVA followed by testing for multivariate dispersion with PERMDISP. To account for inter- and intra-patient variability, PERMANOVA and PERMDISP were performed on profiles averaged across implants with the same diagnosis from the same patient. Diversity indices were calculated using DIVERSE. CAP was used to identify features correlated with disease groups. Differential expression analysis was conducted using DESeq2 (111) and controlling for patients, with significant features (log2 fold change > 1, adjusted *P* < 0.05). Functional pathway analysis was performed using KEGG Mapper for bacterial enzymes (112) and Gene Set Enrichment Analysis (GSEA) for host genes (113). For univariate comparisons, the Kruskal–Wallis test was applied with *post hoc* Dunn’s or Wilcoxon rank-sum test followed by Benjamini–Hochberg correction. For integrative analysis across 16S sequencing (species level), metatranscriptomic bacterial (MTX-B), host (MTX-H), and phage (MTX-P) data sets, samples were standardized, filtered to retain features >0.1% in at least one sample, and log(*x* + 1)-transformed. Significant associations (FDR-adjusted *P* < 0.05) were identified using hierarchical all-against-all association testing (HAllA) (114).

## Data Availability

All raw sequencing data are deposited to ENA under the Bioproject ID PRJNA1192962. The STORMS checklist for this study can be accessed via https://doi.org/10.5281/zenodo.21359485.

## SUPPLEMENTAL MATERIAL

File S1 msystems.00754-25-s0001.xlsx Tables S2–S9.

File S2 msystems.00754-25-s0002.xlsx Tables S10–S15.

Supplemental Information msystems.00754-25-s0003.docx Supplemental methods, figures, and table captions; Table S1.

ASM does not own the copyrights to Supplemental Material that may be linked to, or accessed through, an article. The authors have granted ASM a non-exclusive, world-wide license to publish the Supplemental Material files. Please contact the corresponding author directly for reuse.

## References

[1] Elani HW, Starr JR, Da Silva JD, Gallucci GO. 2018. Trends in dental implant use in the U.S., 1999–2016, and projections to 2026. J Dent Res 97:1424–1430. doi:10.1177/0022034518792567 30075090 PMC6854267

[2] Derks J, Tomasi C. 2015. Peri-implant health and disease. A systematic review of current epidemiology. J Clin Periodontol 42:S158–S171. doi:10.1111/jcpe.12334 25495683

[3] Dreyer H, Grischke J, Tiede C, Eberhard J, Schweitzer A, Toikkanen SE, Glöckner S, Krause G, Stiesch M. 2018. Epidemiology and risk factors of peri-implantitis: a systematic review. J Periodontal Res 53:657–681. doi:10.1111/jre.12562 29882313

[4] Saleh MHA, Dias DR, Kumar P. 2025. The economic and societal impact of periodontal and peri-implant diseases. Periodontol 2000 98:100–118. doi:10.1111/prd.12568 38693603 PMC12842886

[5] Shiba T, Watanabe T, Kachi H, Koyanagi T, Maruyama N, Murase K, Takeuchi Y, Maruyama F, Izumi Y, Nakagawa I. 2016. Distinct interacting core taxa in co-occurrence networks enable discrimination of polymicrobial oral diseases with similar symptoms. Sci Rep 6:30997. doi:10.1038/srep30997 27499042 PMC4976368

[6] Schincaglia GP, Hong BY, Rosania A, Barasz J, Thompson A, Sobue T, Panagakos F, Burleson JA, Dongari-Bagtzoglou A, Diaz PI. 2017. Clinical, immune, and microbiome traits of gingivitis and peri-implant mucositis. J Dent Res 96:47–55. doi:10.1177/0022034516668847 28033066

[7] Ghensi P, Manghi P, Zolfo M, Armanini F, Pasolli E, Bolzan M, Bertelle A, Dell’Acqua F, Dellasega E, Waldner R, Tessarolo F, Tomasi C, Segata N. 2020. Strong oral plaque microbiome signatures for dental implant diseases identified by strain-resolution metagenomics. NPJ Biofilms Microbiomes 6:47. doi:10.1038/s41522-020-00155-7 33127901 PMC7603341

[8] Belibasakis GN, Manoil D. 2021. Microbial community-driven etiopathogenesis of peri-implantitis. J Dent Res 100:21–28. doi:10.1177/0022034520949851 32783779 PMC7754824

[9] Carvalho ÉBS, Romandini M, Sadilina S, Sant’Ana ACP, Sanz M. 2023. Microbiota associated with peri-implantitis-a systematic review with meta-analyses. Clin Oral Implants Res 34:1176–1187. doi:10.1111/clr.14153 37523470

[10] Li S, Sun F, Wei Y, Nie Y, Wu X, Hu W. 2023. Mucosal bleeding correlates with submucosal microbial dysbiosis in peri-implant mucositis of patients with periodontitis. Clinical Oral Implants Res 34:947–957. doi:10.1111/clr.14120 37358250

[11] Feng Z, Zhu J, Zhang L, Li C, Su D, Wang H, Yu Y, Song L. 2024. Microbiological and functional traits of peri-implant mucositis and correlation with disease severity. mSphere 9:e00059-24. doi:10.1128/msphere.00059-24 38980075 PMC11287996

[12] Bazzani D, Heidrich V, Manghi P, Blanco-Miguez A, Asnicar F, Armanini F, Cavaliere S, Bertelle A, Dell’Acqua F, Dellasega E, Waldner R, Vicentini D, Bolzan M, Tomasi C, Segata N, Pasolli E, Ghensi P. 2024. Favorable subgingival plaque microbiome shifts are associated with clinical treatment for peri-implant diseases. NPJ Biofilms Microbiomes 10:12. doi:10.1038/s41522-024-00482-z 38374114 PMC10876967

[13] Dutra TP, Freitas Monteiro M, França-Grohmann IL, Casarin RCV, Casati MZ, Silvério Ruiz KG, Kumar PS, Sallum EA. 2024. Clinical, immunological and microbiological evaluation of experimental peri-implant mucositis and gingivitis in subjects with grade C, stage III/IV periodontitis background. J Clin Periodontol 51:209–221. doi:10.1111/jcpe.13896 37941050

[14] Yu P, Tu C, Wara‐aswapati N, Wang C, Tu Y, Hou H, Ueno T, Chen I, Fu K, Li H, Chen Y. 2024. Microbiome of periodontitis and peri-implantitis before and after therapy: long-read 16S rRNA gene amplicon sequencing. J Periodontal Res 59:657–668. doi:10.1111/jre.13269 38718089

[15] Alves CH, Russi KL, Rocha NC, Bastos F, Darrieux M, Parisotto TM, Girardello R. 2022. Host-microbiome interactions regarding peri-implantitis and dental implant loss. J Transl Med 20:425. doi:10.1186/s12967-022-03636-9 36138430 PMC9502891

[16] Ganesan SM, Dabdoub SM, Nagaraja HN, Mariotti AJ, Ludden CW, Kumar PS. 2022. Biome-microbiome interactions in peri-implantitis: a pilot investigation. J Periodontol 93:814–823. doi:10.1002/JPER.21-0423 35073418 PMC9187590

[17] Robitaille N, Reed DN, Walters JD, Kumar PS. 2016. Periodontal and peri-implant diseases: identical or fraternal infections? Mol Oral Microbiol 31:285–301. doi:10.1111/omi.12124 26255984

[18] Socransky SS, Haffajee AD, Cugini MA, Smith C, Kent RL. 1998. Microbial complexes in subgingival plaque. J Clin Periodontol 25:134–144. doi:10.1111/j.1600-051X.1998.tb02419.x 9495612

[19] Hong B-Y, Furtado Araujo MV, Strausbaugh LD, Terzi E, Ioannidou E, Diaz PI. 2015. Microbiome profiles in periodontitis in relation to host and disease characteristics. PLoS One 10:e0127077. doi:10.1371/journal.pone.0127077 25984952 PMC4436126

[20] Schwarz F, Derks J, Monje A, Wang HLP. 2018. Peri-implantitis. J Clin Periodontol 45:S246–S266. doi:10.1111/jcpe.12954 29926484

[21] Shokeen B, Dinis MDB, Haghighi F, Tran NC, Lux R. 2021. Omics and interspecies interaction. Periodontol 2000 85:101–111. doi:10.1111/prd.12354 33226675

[22] Baker JL. 2023. Illuminating the oral microbiome and its host interactions: recent advancements in omics and bioinformatics technologies in the context of oral microbiome research. FEMS Microbiol Rev 47:fuad051. doi:10.1093/femsre/fuad051 37667515 PMC10503653

[23] Rajagopala SV, Bakhoum NG, Pakala SB, Shilts MH, Rosas-Salazar C, Mai A, Boone HH, McHenry R, Yooseph S, Halasa N, Das SR. 2021. Metatranscriptomics to characterize respiratory virome, microbiome, and host response directly from clinical samples. Cell Rep Methods 1:100091. doi:10.1016/j.crmeth.2021.100091 34790908 PMC8594859

[24] Ren L, Zhang R, Rao J, Xiao Y, Zhang Z, Yang B, Cao D, Zhong H, Ning P, Shang Y, Li M, Gao Z, Wang J. 2018. Transcriptionally active lung microbiome and its association with bacterial biomass and host inflammatory status. mSystems 3:e00199-18. doi:10.1128/mSystems.00199-18 30417108 PMC6208642

[25] Lloyd-Price J, Arze C, Ananthakrishnan AN, Schirmer M, Avila-Pacheco J, Poon TW, Andrews E, Ajami NJ, Bonham KS, Brislawn CJ, et al.. 2019. Multi-omics of the gut microbial ecosystem in inflammatory bowel diseases. Nature 569:655–662. doi:10.1038/s41586-019-1237-9 31142855 PMC6650278

[26] Worby CJ, Schreiber HL IV, Straub TJ, van Dijk LR, Bronson RA, Olson BS, Pinkner JS, Obernuefemann CLP, Muñoz VL, Paharik AE, Azimzadeh PN, Walker BJ, Desjardins CA, Chou W-C, Bergeron K, Chapman SB, Klim A, Manson AL, Hannan TJ, Hooton TM, Kau AL, Lai HH, Dodson KW, Hultgren SJ, Earl AM. 2022. Longitudinal multi-omics analyses link gut microbiome dysbiosis with recurrent urinary tract infections in women. Nat Microbiol 7:630–639. doi:10.1038/s41564-022-01107-x 35505248 PMC9136705

[27] Yan Z, Chen B, Yang Y, Yi X, Wei M, Ecklu-Mensah G, Buschmann MM, Liu H, Gao J, Liang W, et al.. 2022. Multi-omics analyses of airway host-microbe interactions in chronic obstructive pulmonary disease identify potential therapeutic interventions. Nat Microbiol 7:1361–1375. doi:10.1038/s41564-022-01196-8 35995842

[28] Long H, Yan L, Pu J, Liu Y, Zhong X, Wang H, Yang L, Lou F, Luo S, Zhang Y, Liu Y, Xie P, Ji P, Jin X. 2022. Multi-omics analysis reveals the effects of microbiota on oral homeostasis. Front Immunol 13:1005992. doi:10.3389/fimmu.2022.1005992 36211346 PMC9533175

[29] Berglundh T, Armitage G, Araujo MG, Avila-Ortiz G, Blanco J, Camargo PM, Chen S, Cochran D, Derks J, Figuero E, Hämmerle CHF, Heitz-Mayfield LJA, Huynh-Ba G, Iacono V, Koo K-T, Lambert F, McCauley L, Quirynen M, Renvert S, Salvi GE, Schwarz F, Tarnow D, Tomasi C, Wang H-L, Zitzmann N. 2018. Peri-implant diseases and conditions: consensus report of workgroup 4 of the 2017 world workshop on the classification of periodontal and peri-implant diseases and conditions. J Clin Periodontol 45:S286–S291. doi:10.1111/jcpe.12957 29926491

[30] Zheng H, Xu L, Wang Z, Li L, Zhang J, Zhang Q, Chen T, Lin J, Chen F. 2015. Subgingival microbiome in patients with healthy and ailing dental implants. Sci Rep 5:10948. doi:10.1038/srep10948 26077225 PMC4468443

[31] Joshi AA, Szafrański SP, Steglich M, Yang I, Qu T, Schaefer-Dreyer P, Xiao X, Behrens W, Grischke J, Häussler S, Stiesch M. 2025. Integrative microbiome- and metatranscriptome-based analyses reveal diagnostic biomarkers for peri-implantitis. NPJ Biofilms Microbiomes 11:175. doi:10.1038/s41522-025-00807-6 40858628 PMC12381052

[32] Moore WE, Moore LH, Ranney RR, Smibert RM, Burmeister JA, Schenkein HA. 1991. The microflora of periodontal sites showing active destructive progression. J Clin Periodontol 18:729–739. doi:10.1111/j.1600-051x.1991.tb00064.x 1752997

[33] Chen T, Yu W-H, Izard J, Baranova OV, Lakshmanan A, Dewhirst FE. 2010. The human oral microbiome database: a web accessible resource for investigating oral microbe taxonomic and genomic information. Database (Oxford) 2010:baq013. doi:10.1093/database/baq013 20624719 PMC2911848

[34] Marsh PD, Zaura E. 2017. Dental biofilm: ecological interactions in health and disease. J Clin Periodontol 44:S12–S22. doi:10.1111/jcpe.12679 28266111

[35] Bowen WH, Burne RA, Wu H, Koo H. 2018. Oral biofilms: pathogens, matrix, and polymicrobial interactions in microenvironments. Trends Microbiol 26:229–242. doi:10.1016/j.tim.2017.09.008 29097091 PMC5834367

[36] Szafrański SP, Slots J, Stiesch M. 2021. The human oral phageome. Periodontol 2000 86:79–96. doi:10.1111/prd.12363 33690937

[37] Imamura T. 2003. The role of gingipains in the pathogenesis of periodontal disease. J Periodontol 74:111–118. doi:10.1902/jop.2003.74.1.111 12593605

[38] Dieckow S, Szafrański SP, Grischke J, Qu T, Doll-Nikutta K, Steglich M, Yang I, Häussler S, Stiesch M. 2024. Structure and composition of early biofilms formed on dental implants are complex, diverse, subject-specific and dynamic. NPJ Biofilms Microbiomes 10:155. doi:10.1038/s41522-024-00624-3 39719447 PMC11668855

[39] Kumar PS, Mason MR, Brooker MR, O’Brien K. 2012. Pyrosequencing reveals unique microbial signatures associated with healthy and failing dental implants. J Clin Periodontol 39:425–433. doi:10.1111/j.1600-051X.2012.01856.x 22417294 PMC3323747

[40] Tsigarida AA, Dabdoub SM, Nagaraja HN, Kumar PS. 2015. The influence of smoking on the peri-implant microbiome. J Dent Res 94:1202–1217. doi:10.1177/0022034515590581 26124222 PMC4547314

[41] Sanz‐Martin I, Doolittle‐Hall J, Teles RP, Patel M, Belibasakis GN, Hämmerle CHF, Jung RE, Teles FRF. 2017. Exploring the microbiome of healthy and diseased peri-implant sites using Illumina sequencing. J Clin Periodontol 44:1274–1284. doi:10.1111/jcpe.12788 28766745 PMC7209775

[42] Kröger A, Hülsmann C, Fickl S, Spinell T, Hüttig F, Kaufmann F, Heimbach A, Hoffmann P, Enkling N, Renvert S, Schwarz F, Demmer RT, Papapanou PN, Jepsen S, Kebschull M. 2018. The severity of human peri-implantitis lesions correlates with the level of submucosal microbial dysbiosis. J Clin Periodontol 45:1498–1509. doi:10.1111/jcpe.13023 30341964

[43] Lamont RJ, Hajishengallis G. 2015. Polymicrobial synergy and dysbiosis in inflammatory disease. Trends Mol Med 21:172–183. doi:10.1016/j.molmed.2014.11.004 25498392 PMC4352384

[44] Monje A, Kan JY, Borgnakke W. 2023. Impact of local predisposing/precipitating factors and systemic drivers on peri-implant diseases. Clin Implant Dent Rel Res 25:640–660. doi:10.1111/cid.13155 36533411

[45] da Silva MK, de Carvalho ACG, Alves EHP, da Silva FRP, Pessoa L dos S, Vasconcelos DFP. 2017. Genetic factors and the risk of periodontitis development: findings from a systematic review composed of 13 studies of meta-analysis with 71,531 participants. Int J Dent 2017:1–9. doi:10.1155/2017/1914073 PMC5424192 28529526

[46] Belstrøm D, Constancias F, Drautz-Moses DI, Schuster SC, Veleba M, Mahé F, Givskov M. 2021. Periodontitis associates with species-specific gene expression of the oral microbiota. NPJ Biofilms Microbiomes 7:76. doi:10.1038/s41522-021-00247-y 34556654 PMC8460658

[47] Belstrøm D, Constancias F, Liu Y, Yang L, Drautz-Moses DI, Schuster SC, Kohli GS, Jakobsen TH, Holmstrup P, Givskov M. 2017. Metagenomic and metatranscriptomic analysis of saliva reveals disease-associated microbiota in patients with periodontitis and dental caries. NPJ Biofilms Microbiomes 3:23. doi:10.1038/s41522-017-0031-4 28979798 PMC5624903

[48] Nowicki EM, Shroff R, Singleton JA, Renaud DE, Wallace D, Drury J, Zirnheld J, Colleti B, Ellington AD, Lamont RJ, Scott DA, Whiteley M. 2018. Microbiota and metatranscriptome changes accompanying the onset of gingivitis. mBio 9:e00575-18. doi:10.1128/mBio.00575-18 29666288 PMC5904416

[49] Szafrański SP, Deng Z-L, Tomasch J, Jarek M, Bhuju S, Meisinger C, Kühnisch J, Sztajer H, Wagner-Döbler I. 2015. Functional biomarkers for chronic periodontitis and insights into the roles of *Prevotella nigrescens* and *Fusobacterium nucleatum*; a metatranscriptome analysis. NPJ Biofilms Microbiomes 1:15017. doi:10.1038/npjbiofilms.2015.17 28721234 PMC5515211

[50] Szafranski SP, Wos-Oxley ML, Vilchez-Vargas R, Jáuregui R, Plumeier I, Klawonn F, Tomasch J, Meisinger C, Kühnisch J, Sztajer H, Pieper DH, Wagner-Döbler I. 2015. High-resolution taxonomic profiling of the subgingival microbiome for biomarker discovery and periodontitis diagnosis. Appl Environ Microbiol 81:1047–1058. doi:10.1128/AEM.03534-14 25452281 PMC4292489

[51] Qu T, Koch L, Mukherjee R, Tu Y, Seidel AL, Püttmann LD, Winkel A, Yang I, Grischke J, Liu D, Wolkers WF, Kittler S, Chichkov B, Stiesch M, Szafrański SP. 2025. Laser-assisted microbial culturomics. Nat Commun 16:10614. doi:10.1038/s41467-025-66804-7 41298564 PMC12661035

[52] Jiang S-S, Chen Y-X, Fang J-Y. 2026. *Fusobacterium nucleatum*: ecology, pathogenesis and clinical implications. Nat Rev Microbiol 24:197–214. doi:10.1038/s41579-025-01237-z 40983729

[53] Perera D, McLean A, Morillo-López V, Cloutier-Leblanc K, Almeida E, Cabana K, Mark Welch J, Ramsey M. 2022. Mechanisms underlying interactions between two abundant oral commensal bacteria. ISME J 16:948–957. doi:10.1038/s41396-021-01141-3 34732850 PMC8940909

[54] Giacomini JJ, Torres-Morales J, Tang J, Dewhirst FE, Borisy GG, Mark Welch JL. 2024. Spatial ecology of *Haemophilus and Aggregatibacter* in the human oral cavity. Microbiol Spectr 12:e04017-23. doi:10.1128/spectrum.04017-23 38488280 PMC10986600

[55] Szafrański SP, Kilian M, Yang I, Bei der Wieden G, Winkel A, Hegermann J, Stiesch M. 2019. Diversity patterns of bacteriophages infecting *Aggregatibacter* and *Haemophilus* species across clades and niches. ISME J 13:2500–2522. doi:10.1038/s41396-019-0450-8 31201356 PMC6776037

[56] Liu G, Tang CM, Exley RM. 2015. Non-pathogenic *Neisseria*: members of an abundant, multi-habitat, diverse genus. Microbiology (Reading) 161:1297–1312. doi:10.1099/mic.0.000086 25814039

[57] Zhou N, Huang H, Liu H, Li Q, Yang G, Zhang Y, Ding M, Dong H, Mou Y. 2022. Microbiota analysis of peri-implant mucositis in patients with periodontitis history. Clin Oral Investig 26:6223–6233. doi:10.1007/s00784-022-04571-1 PMC9525361 35672515

[58] Wang M, Liu YB, Tong WM, Leung WK, He LL, Xu X, Xu D, Zhou Q. 2025. Periodontitis history shapes the early peri-implant microbiome formation: a metagenomic analysis. J Clin Periodontol 52:1011–1023. doi:10.1111/jcpe.14147 40043731 PMC12176463

[59] Li L, Mac Aogáin M, Xu T, Jaggi TK, Chan LLY, Qu J, Wei L, Liao S, Cheng HS, Keir HR, et al.. 2022. *Neisseria* species as pathobionts in bronchiectasis. Cell Host Microbe 30:1311–1327. doi:10.1016/j.chom.2022.08.005 36108613

[60] Toney AM, Koirala J, Stacy A. 2025. Metabolic strategies that enable oral commensal persistence in a lower airway environment. mBio 16:e01948-25. doi:10.1128/mbio.01948-25 40980886 PMC12607641

[61] Criss AK, Seifert HS. 2012. A bacterial siren song: intimate interactions between *Neisseria* and neutrophils. Nat Rev Microbiol 10:178–190. doi:10.1038/nrmicro2713 22290508 PMC3569855

[62] Giacomini JJ, Torres-Morales J, Dewhirst FE, Borisy GG, Mark Welch JL. 2025. Spatial ecology of the *Neisseriaceae* family in the human oral cavity. Microbiol Spectr 13:e03275-24. doi:10.1128/spectrum.03275-24 40197060 PMC12054151

[63] Bennett JS, Watkins ER, Jolley KA, Harrison OB, Maiden MCJ. 2014. Identifying *Neisseria* species by use of the 50S ribosomal protein L6 (*rplF*) gene. J Clin Microbiol 52:1375–1381. doi:10.1128/JCM.03529-13 24523465 PMC3993661

[64] Bennett J.S., Jolley KA, Maiden MCJ. 2013. Genome sequence analyses show that *Neisseria oralis* is the same species as ' *Neisseria mucosa* var. *heidelbergensis*'. Int J Syst Evol Microbiol 63:3920–3926. doi:10.1099/ijs.0.052431-0 24097834 PMC3799226

[65] Perkins-Balding D, Ratliff-Griffin M, Stojiljkovic I. 2004. Iron transport systems in *Neisseria meningitidis*. Microbiol Mol Biol Rev 68:154–171. doi:10.1128/MMBR.68.1.154-171.2004 15007100 PMC362107

[66] Rosier BT, Buetas E, Moya-Gonzalvez EM, Artacho A, Mira A. 2020. Nitrate as a potential prebiotic for the oral microbiome. Sci Rep 10:12895. doi:10.1038/s41598-020-69931-x 32732931 PMC7393384

[67] Morou-Bermúdez E, Torres-Colón JE, Bermúdez NS, Patel RP, Joshipura KJ. 2022. Pathways linking oral bacteria, nitric oxide metabolism, and health. J Dent Res 101:623–631. doi:10.1177/00220345211064571 35081826 PMC9124908

[68] Zitzmann NU, Berglundh T, Marinello CP, Lindhe J. 2001. Experimental peri-implant mucositis in man. J Clinic Periodontology 28:517–523. doi:10.1034/j.1600-051x.2001.028006517.x 11350518

[69] Yost S, Duran-Pinedo AE, Teles R, Krishnan K, Frias-Lopez J. 2015. Functional signatures of oral dysbiosis during periodontitis progression revealed by microbial metatranscriptome analysis. Genome Med 7:27. doi:10.1186/s13073-015-0153-3 25918553 PMC4410737

[70] Miller DP, Fitzsimonds ZR, Lamont RJ. 2019. Metabolic signaling and spatial interactions in the oral polymicrobial community. J Dent Res 98:1308–1314. doi:10.1177/0022034519866440 31356756 PMC6806133

[71] Hojo K, Nagaoka S, Ohshima T, Maeda N. 2009. Bacterial interactions in dental biofilm development. J Dent Res 88:982–990. doi:10.1177/0022034509346811 19828884

[72] Kuramitsu HK, He X, Lux R, Anderson MH, Shi W. 2007. Interspecies interactions within oral microbial communities. Microbiol Mol Biol Rev 71:653–670. doi:10.1128/MMBR.00024-07 18063722 PMC2168648

[73] Zhang M, Whiteley M, Lewin GR. 2022. Polymicrobial interactions of oral microbiota: a historical review and current perspective. mBio 13:e0023522. doi:10.1128/mbio.00235-22 35491817 PMC9239150

[74] Diaz PI, Valm AM. 2020. Microbial interactions in oral communities mediate emergent biofilm properties. J Dent Res 99:18–25. doi:10.1177/0022034519880157 31590609 PMC6927214

[75] Dahlman S, Avellaneda-Franco L, Rutten EL, Gulliver EL, Solari S, Chonwerawong M, Kett C, Subedi D, Young RB, Campbell N, Gould JA, Bell JD, Docherty CAH, Turkington CJR, Nezam-Abadi N, Grasis JA, Lyras D, Edwards RA, Forster SC, Barr JJ. 2025. Isolation, engineering and ecology of temperate phages from the human gut. Nature 647:698–705. doi:10.1038/s41586-025-09614-7 41094135 PMC12629997

[76] Vartoukian SR, Adamowska A, Lawlor M, Moazzez R, Dewhirst FE, Wade WG. 2016. *In vitro* cultivation of 'unculturable' oral bacteria, facilitated by community culture and media supplementation with siderophores. PLoS One 11:e0146926. doi:10.1371/journal.pone.0146926 26764907 PMC4713201

[77] Deng Z-L, Szafrański SP, Jarek M, Bhuju S, Wagner-Döbler I. 2017. Dysbiosis in chronic periodontitis: key microbial players and interactions with the human host. Sci Rep 7:3703. doi:10.1038/s41598-017-03804-8 28623321 PMC5473847

[78] Lin G-H, Chan H-L, Wang H-L. 2013. The significance of keratinized mucosa on implant health: a systematic review. J Periodontol 84:1755–1767. doi:10.1902/jop.2013.120688 23451989

[79] Ramanauskaite A, Schwarz F, Sader R. 2022. Influence of width of keratinized tissue on the prevalence of peri-implant diseases: a systematic review and meta-analysis. Clin Oral Implants Res 33:8–31. doi:10.1111/clr.13766 35763022

[80] Page RC. 1991. The role of inflammatory mediators in the pathogenesis of periodontal disease. J Periodontal Res 26:230–242. doi:10.1111/j.1600-0765.1991.tb01649.x 1679130

[81] Cekici A, Kantarci A, Hasturk H, Van Dyke TE. 2014. Inflammatory and immune pathways in the pathogenesis of periodontal disease. Periodontol 2000 64:57–80. doi:10.1111/prd.12002 24320956 PMC4500791

[82] Darveau RP, Pham T-TT, Lemley K, Reife RA, Bainbridge BW, Coats SR, Howald WN, Way SS, Hajjar AM. 2004. *Porphyromonas gingivalis* lipopolysaccharide contains multiple lipid A species that functionally interact with both toll-like receptors 2 and 4. Infect Immun 72:5041–5051. doi:10.1128/IAI.72.9.5041-5051.2004 15321997 PMC517442

[83] Chandel NS, Trzyna WC, McClintock DS, Schumacker PT. 2000. Role of oxidants in NF-κB activation and TNF-α gene transcription induced by hypoxia and endotoxin. J Immunol 165:1013–1021. doi:10.4049/jimmunol.165.2.1013 10878378

[84] Marcinkiewicz J, Kontny E. 2014. Taurine and inflammatory diseases. Amino Acids 46:7–20. doi:10.1007/s00726-012-1361-4 22810731 PMC3894431

[85] Sturman JA. 1993. Taurine in development. Physiol Rev 73:119–147. doi:10.1152/physrev.1993.73.1.119 8419963

[86] Abusleme L, Dupuy AK, Dutzan N, Silva N, Burleson JA, Strausbaugh LD, Gamonal J, Diaz PI. 2013. The subgingival microbiome in health and periodontitis and its relationship with community biomass and inflammation. ISME J 7:1016–1025. doi:10.1038/ismej.2012.174 23303375 PMC3635234

[87] Cai Z, Lin S, Hu S, Zhao L. 2021. Structure and function of oral microbial community in periodontitis based on integrated data. Front Cell Infect Microbiol 11:663756. doi:10.3389/fcimb.2021.663756 34222038 PMC8248787

[88] Iniesta M, Chamorro C, Ambrosio N, Marín MJ, Sanz M, Herrera D. 2023. Subgingival microbiome in periodontal health, gingivitis and different stages of periodontitis. J Clin Periodontol 50:905–920. doi:10.1111/jcpe.13793 36792073

[89] Colombo APV, Tanner ACR. 2019. The role of bacterial biofilms in dental caries and periodontal and peri-implant diseases: a historical perspective. J Dent Res 98:373–385. doi:10.1177/0022034519830686 30890060

[90] Cross KL, Chirania P, Xiong W, Beall CJ, Elkins JG, Giannone RJ, Griffen AL, Guss AM, Hettich RL, Joshi SS, Mokrzan EM, Martin RK, Zhulin IB, Leys EJ, Podar M. 2018. Insights into the evolution of host association through the isolation and characterization of a novel human periodontal pathobiont, *Desulfobulbus oralis*. mBio 9:e02061-17. doi:10.1128/mBio.02061-17 29535201 PMC5850319

[91] Campbell AG, Schwientek P, Vishnivetskaya T, Woyke T, Levy S, Beall CJ, Griffen A, Leys E, Podar M. 2014. Diversity and genomic insights into the uncultured *Chloroflexi* from the human microbiota. Environ Microbiol 16:2635–2643. doi:10.1111/1462-2920.12461 24738594 PMC4149597

[92] Bernstein DB, Dewhirst FE, Segrè D. 2019. Metabolic network percolation quantifies biosynthetic capabilities across the human oral microbiome. eLife 8:e39733. doi:10.7554/eLife.39733 31194675 PMC6609349

[93] Langendijk PS, Hanssen JT, Van der Hoeven JS. 2000. Sulfate-reducing bacteria in association with human periodontitis. J Clin Periodontol 27:943–950. doi:10.1034/j.1600-051x.2000.027012943.x 11140562

[94] Hill GB, Ayers OM, Kohan AP. 1987. Characteristics and sites of infection of *Eubacterium nodatum*, *Eubacterium timidum*, *Eubacterium brachy*, and other asaccharolytic eubacteria. J Clin Microbiol 25:1540–1545. doi:10.1128/jcm.25.8.1540-1545.1987 3624445 PMC269265

[95] Langendijk PS, Kulik EM, Sandmeier H, Meyer J, van der Hoeven JS. 2001. Isolation of *Desulfomicrobium orale* sp. nov. and *Desulfovibrio* strain NY682, oral sulfate-reducing bacteria involved in human periodontal disease. Int J Syst Evol Microbiol 51:1035–1044. doi:10.1099/00207713-51-3-1035 11411671

[96] Loubinoux J, Bisson-Boutelliez C, Miller N, Le Faou AE. 2002. Isolation of the provisionally named *Desulfovibrio fairfieldensis* from human periodontal pockets. Oral Microbiol Immunol 17:321–323. doi:10.1034/j.1399-302x.2002.170510.x 12354215

[97] Ye H, Borusak S, Eberl C, Krasenbrink J, Weiss AS, Chen S-C, Hanson BT, Hausmann B, Herbold CW, Pristner M, Zwirzitz B, Warth B, Pjevac P, Schleheck D, Stecher B, Loy A. 2023. Ecophysiology and interactions of a taurine-respiring bacterium in the mouse gut. Nat Commun 14:5533. doi:10.1038/s41467-023-41008-z 37723166 PMC10507020

[98] Dar D, Dar N, Cai L, Newman DK. 2021. Spatial transcriptomics of planktonic and sessile bacterial populations at single-cell resolution. Science 373:eabi4882. doi:10.1126/science.abi4882 34385369 PMC8454218

[99] Mark Welch JL, Rossetti BJ, Rieken CW, Dewhirst FE, Borisy GG. 2016. Biogeography of a human oral microbiome at the micron scale. Proc Natl Acad Sci USA 113:E791–E800. doi:10.1073/pnas.1522149113 26811460 PMC4760785

[100] Ingendoh-Tsakmakidis A, Mikolai C, Winkel A, Szafrański SP, Falk CS, Rossi A, Walles H, Stiesch M. 2019. Commensal and pathogenic biofilms differently modulate peri-implant oral mucosa in an organotypic model. Cell Microbiol 21:e13078. doi:10.1111/cmi.13078 31270923 PMC6771885

[101] Doll-Nikutta K, Mikolai C, Heine N, Kurselis K, Fadeeva E, Debener N, Legutko B, Kreuzkamp C, Prasanthan V, Bahnemann J, Chichkov BN, Stiesch M. 2025. Biocompatible liquid-infused titanium minimizes oral biofilm adhesion in flow chamber and 3D implant-tissue-biofilm *in vitro* models. Bioact Mater 53:706–717. doi:10.1016/j.bioactmat.2025.07.048 40801021 PMC12341519

[102] Belibasakis GN, Schmidlin PR, Sahrmann P. 2014. Molecular microbiological evaluation of subgingival biofilm sampling by paper point and curette. APMIS 122:347–352. doi:10.1111/apm.12151 23879704

[103] Grischke J, Szafrański SP, Muthukumarasamy U, Haeussler S, Stiesch M. 2021. Removable denture is a risk indicator for peri-implantitis and facilitates expansion of specific periodontopathogens: a cross-sectional study. BMC Oral Health 21:173. doi:10.1186/s12903-021-01529-9 33794847 PMC8017824

[104] Dewhirst FE, Chen T, Izard J, Paster BJ, Tanner ACR, Yu W-H, Lakshmanan A, Wade WG. 2010. The human oral microbiome. J Bacteriol 192:5002–5017. doi:10.1128/JB.00542-10 20656903 PMC2944498

[105] Pasolli E, Asnicar F, Manara S, Zolfo M, Karcher N, Armanini F, Beghini F, Manghi P, Tett A, Ghensi P, Collado MC, Rice BL, DuLong C, Morgan XC, Golden CD, Quince C, Huttenhower C, Segata N. 2019. Extensive unexplored human microbiome diversity revealed by over 150,000 genomes from metagenomes spanning age, geography, and lifestyle. Cell 176:649–662. doi:10.1016/j.cell.2019.01.001 30661755 PMC6349461

[106] Huerta-Cepas J, Szklarczyk D, Heller D, Hernández-Plaza A, Forslund SK, Cook H, Mende DR, Letunic I, Rattei T, Jensen LJ, von Mering C, Bork P. 2019. eggNOG 5.0: a hierarchical, functionally and phylogenetically annotated orthology resource based on 5090 organisms and 2502 viruses. Nucleic Acids Res 47:D309–D314. doi:10.1093/nar/gky1085 30418610 PMC6324079

[107] Jorth P, Turner KH, Gumus P, Nizam N, Buduneli N, Whiteley M. 2014. Metatranscriptomics of the human oral microbiome during health and disease. mBio 5:e01012-14. doi:10.1128/mBio.01012-14 24692635 PMC3977359

[108] Roux S, Páez-Espino D, Chen I-M, Palaniappan K, Ratner A, Chu K, Reddy TBK, Nayfach S, Schulz F, Call L, Neches RY, Woyke T, Ivanova NN, Eloe-Fadrosh EA, Kyrpides NC. 2021. IMG/VR v3: an integrated ecological and evolutionary framework for interrogating genomes of uncultivated viruses. Nucleic Acids Res 49:D764–D775. doi:10.1093/nar/gkaa946 33137183 PMC7778971

[109] Anderson M, Gorley RN, Clarke K. 2008. PERMANOVA+ for primer: guide to software and statistical methods.

[110] Clarke K, Gorley R. 2015. PRIMER version 7: user manual/tutorial. PRIMER-E 192.

[111] Love MI, Huber W, Anders S. 2014. Moderated estimation of fold change and dispersion for RNA-seq data with DESeq2. Genome Biol 15:550. doi:10.1186/s13059-014-0550-8 25516281 PMC4302049

[112] Kanehisa M, Sato Y, Kawashima M. 2022. KEGG mapping tools for uncovering hidden features in biological data. Protein Sci 31:47–53. doi:10.1002/pro.4172 34423492 PMC8740838

[113] Subramanian A, Tamayo P, Mootha VK, Mukherjee S, Ebert BL, Gillette MA, Paulovich A, Pomeroy SL, Golub TR, Lander ES, Mesirov JP. 2005. Gene set enrichment analysis: a knowledge-based approach for interpreting genome-wide expression profiles. Proc Natl Acad Sci USA 102:15545–15550. doi:10.1073/pnas.0506580102 16199517 PMC1239896

[114] Ghazi AR, Sucipto K, Rahnavard A, Franzosa EA, McIver LJ, Lloyd-Price J, Schwager E, Weingart G, Moon YS, Morgan XC, Waldron L, Huttenhower C. 2022. High-sensitivity pattern discovery in large, paired multiomic datasets. Bioinformatics 38:i378–i385. doi:10.1093/bioinformatics/btac232 35758795 PMC9235493

