# Supplementary material for: High-resolution taxonomic profiling and metatranscriptomics identify microbial, biochemical, host, and ecological factors in peri-implant disease
Source: mSystems. 2026 Jul 31;11(8):e00754-25. doi: 10.1128/msystems.00754-25 (PMC13483434; doi:10.1128/msystems.00754-25)
Supplement: Supplementary file 1 [file msystems.00754-25-s0003.docx]

# Supplementary Information: High-resolution taxonomic profiling and metatranscriptomics identify microbial, biochemical, host and ecological factors in peri-implant disease

Short title: Peri-implant ecosystem; SI

Szymon P. Szafrański^1,2,3,$,*^, Amruta A. Joshi^1,2,*^, Matthias Steglich^1,2^, Ines Yang^1,2^, Taoran Qu^1,2^, Wiebke Behrens^1,2^, Uthayakumar Muthukumarasamy^4^, Damianos Melidis^5^, Paula Schaefer-Dreyer^1^, Jasmin Grischke^1^, Jan Hegermann^6^, Wolfgang Nejdl^5,7^, Susanne Häussler^3,4,8,9^, and Meike Stiesch^1,2,3,$^

^1^Department of Prosthetic Dentistry and Biomedical Materials Science, Hannover Medical School, Hannover, Germany

²Lower Saxony Centre for Biomedical Engineering, Implant Research and Development (NIFE), Hannover, Germany

^3^Cluster of Excellence RESIST (EXC 2155), Hannover Medical School, Hannover, Germany

^4^Department of Molecular Bacteriology, Helmholtz Centre for Infection Research, Braunschweig, Germany

^5^L3S Research Center, Leibniz University Hannover, Hannover, Germany

^6^Research Core Unit Electron Microscopy, Institute of Functional and Applied Anatomy, Hannover Medical School, Hannover, Germany

^7^Knowledge-based Systems Laboratory, Leibniz University Hannover, Hannover, Germany

^8^Institute for Molecular Bacteriology, Twincore, Centre for Clinical and Experimental Infection Research, Hannover, Germany.

^9^Department of Clinical Microbiology, Copenhagen University Hospital - Rigshospitalet, Copenhagen, Denmark

*Szymon P. Szafrański and Amruta A. Joshi contributed equally

^$^correspondence to: Meike Stiesch: Email: Stiesch.Meike@mh-hannover.de (lead contact) and Szymon P. Szafrański: Email: Szafranski.Szymon@mh-hannover.de

# Supplementary Methods

## Clinical diagnosis and examination

Peri-implant health and disease diagnosis was based on the 2017 World Workshop on the Classification of Periodontal and Peri-implant Diseases and Conditions^1^. Accordingly, peri-implant health (PIH) was characterized by the absence of the following: clinical signs of inflammation, bleeding and/or suppuration, increased probing depth (PD) compared to previous examinations, or bone loss beyond crestal bone level changes due initial bone remodeling. Peri-implantitis mucositis (PIM) was diagnosed by the presence of bleeding and/or suppuration on gentle probing without the evidence of bone loss. The predefined criteria for peri-implantitis included presence of bleeding and/or suppuration on probing, increased PD and radiographic signs of bone loss as compared to previous examinations, or PD ≥6 mm and bone loss ≥3 mm in absence of previous examinations. The inclusion criteria for this study involved patients over 18 years of age who were systemically healthy, had at least one implant diagnosed with either PIH, PIM or PI. Patients with presence of periodontal disease, uncontrolled diabetes, heavy smoking (>20 cigarettes/day), recent use of antimicrobials or anti-inflammatory medications (within the last six months), pregnancy or lactation, conditions requiring pre-medication before dental procedures, or prior treatment for peri-implant disease on the same implant (except for supragingival prophylaxis) were excluded.

A single experienced clinician recorded detailed medical and dental histories and conducted chair-side examinations for all participants. The patient-level information including age, gender, history of periodontitis and smoking status were recorded, followed by full mouth examination to assess presence of periodontitis, residual teeth and oral hygiene maintenance. For each implant included in the study, clinical parameters including bleeding on probing (BOP), peri-implant probing depth (PD), suppuration, gingival index (GI), plaque index (PI), periotron values type of superstructure (fixed or removable) and duration of implant in function were recorded. Population-specific demographics and implant-specific clinical characteristics are presented in **Supplemental Table 1**.

## Biofilm sample collection and DNA-RNA co-isolation

To efficiently collect biofilm samples and obtain both DNA and RNA from low-biomass samples while preventing RNA degradation and ensuring effective biofilm cell lysis, a tailored column-based protocol was employed, adapted from a previously published method^2^^,^ ^3^. For sampling, sterile paper points (ISO 35/2.0, VDW GmbH, München, Germany) were inserted into the peri-implant sulcus or pocket at six different surfaces (mesio-buccal, mesial, mesio-lingual, disto-lingual, distal and disto-buccal) for 30 sec each, after isolating and drying the cervical region using cotton pellets. Additionally, subgingival plaque was collected at these sites using periodontal curette (HuFriedy Mfg. Co. LLC, Chicago, USA) and was transferred to the paper points before pooling them for each implant. Samples were incubated in RNAprotect (Qiagen, Hilden, Germany) for 5 min at room temperature. The samples were then immediately stored at -80 °C until further processing.

RNA isolation from the biofilm samples was performed as per previous protocol^2^. The protocol was modified to include co-isolation of DNA from the same samples for full-length 16S rRNA gene amplicon sequencing. The optimized protocol addressed key challenges associated with low-biomass biofilm samples, such as efficient lysis and prevention of RNA degradation as well as contamination. Chemicals were obtained from Sigma-Aldrich (Sigma-Aldrich, Taufkirchen, Germany) and the kits were used according to the manufacturer’s instructions, if not stated otherwise. All glassware and other instruments were RNase-decontaminated using RNase ZAP solution (Ambion, Austin, TX, USA). The paper points were thawed and shredded with sterile scissors. The fragmented paper points were incubated in lysis buffer containing 10 mM Tris, 1 mM EDTA, pH 8.0, 2.5 mg/ml lysozyme and 50 U/ml mutanolysin at 25 °C for 1.5 h on a shaking incubator at 350 r.p.m. A total 700 μl of fresh buffer RLT (Qiagen) containing 1% (v/v) β-mercaptoethanol was added and vortexed for 10 sec. Samples (including the fragmented paper points) were placed on a QIAshredder Mini Spin column (Qiagen) and centrifuged for 1 min at 11,000 r.p.m. The flow-through containing the bacterial cells was mixed with 150 mg acid-washed and autoclaved glass beads (diameter 106 μm). Samples were vortexed 10 times for 30 sec at full speed with at least 1-min intervals on ice in between vortexing. The samples were then centrifuged for 1 min at maximal speed. Total RNA was isolated from the supernatants using the RNeasy Mini Kit (Qiagen). DNA was removed by column digestion and by DNase digestion in the eluate using the RNeasy cleanup procedure. Once the eluate for RNA extraction had been separated, DNA isolation was performed by placing the RNAeasy minispin columns in new tubes and incubated with 30 µl of 8 mM NaOH at 55 °C for 10 min. Eluate was collected by centrifugation and 3.03 µl 0.1M HEPES was added to it and both total RNA and DNA were stored at -80 °C until further use.

Blank controls were included to detect nucleic acid contamination due to reagents and paper points. Nucleic acid quality was assessed using the Agilent 2100 Bioanalyzer. To meet sequencing requirements, RNA samples with low yield but from the same patient and with same diagnosis were pooled, resulting in 24 pooled RNA samples from 55 healthy samples.

## Full 16S rRNA gene amplicons sequencing and analysis

Full-length 16S rRNA gene amplicon sequencing (full-16S) was performed on a PacBio Sequel system, followed by bioinformatic analysis using an in-house pipeline^4^. In brief, the full-length 16S rRNA gene from each sample was amplified using the universal primer pair 27F (AGRGTTYGATYMTGGCTCAG) and 1492R (RGYTACCTTGTTACGACTT). PCR amplification was carried out with the KAPA PCR mix for 23 to 27 cycles under the following thermal conditions: an initial denaturation at 95 °C for 30 seconds, annealing at 55 °C for 30 seconds, and extension at 72 °C for 90 seconds, followed by a final elongation step of 10 minutes at 72 °C. After amplification, DNA concentration and quality were assessed using the Invitrogen Qubit dsDNA BR Assay Kit and the Qubit 2.0 fluorometer (Thermo Fisher Scientific). PCR products were then purified using the AMPure bead protocol, and samples with DNA concentrations of 5 ng or higher were selected for subsequent PacBio sequencing (PacBio Biosciences Inc., California, USA). SMRTbell libraries were constructed from the purified amplicons as per the manufacturer’s protocol. Finally, circular consensus sequence (CCS) reads were generated from the raw PacBio data using the manufacturer’s standard software suite.

PacBio CCS sequences were processed using an in-house pipeline^4^^,^ ^5^. Taxonomic identification to the species level was achieved by comparing sequences against a modified bacteria-only version of the SILVA SSU Ref_NR 99 database version 132 ^6^, which was further enriched with Human Oral Microbiome Database (HOMD)_16S_rRNA RefSeq Version 15.1 sequences^7^, and the All-Species Living Tree Project (LTP) database version LTPs132_SSU^8^, into which additional unnamed and phylotype sequences from HOMD 16S_rRNA RefSeq were additionally included. Taxonomic assignments were manually curated. Sequences that could not be confidently assigned to a single species were clustered into operational taxonomic units (OTUs) at 97% identity using UPARSE^9^ as implemented in USEARCH 10.0.240. For higher-level taxonomic classification, the RDP classifier version 2.13 was employed at a cutoff bootstrap confidence of 80%^10^. To ensure data quality, sequences identified as potential contaminants, based on negative controls (blanks), literature information^11^, and correlation analyses^12^ were removed.

For class-level 16S rRNA gene amplicon analyses, bacterial classes were grouped by oxygen tolerance (aerotolerant vs. obligately anaerobic or other) based on established physiological classifications in Bergey’s Manual of Systematic Bacteriology. Category-level scores were computed by summing relative abundances within each group and expressing them as the log_2_(anaerobic/aerotolerant) ratio, which served as the primary metric to avoid redundancy inherent to compositional data. Differences between peri-implant health and peri-implantitis were evaluated using linear mixed-effects models, with diagnosis as a fixed effect and individual as a random intercept to account for multiple implants per subject. Model-derived *P* values were obtained via Type III analysis of variance with Satterthwaite’s approximation for degrees of freedom, as implemented in the lmerTest package. All analyses were performed in R using tidyverse (v2.0.0), lme4 (v1.1-37), lmerTest (v3.1-3), and emmeans (v1.11.1).

An additional amplicon sequence variant (ASV) analysis was conducted as previously described^4^, using FASTQ reads (minimum quality score Q30) derived from the same raw sequencing dataset. Briefly, reads were demultiplexed and primer sequences were removed using tagcleaner. Processing was performed in R with the dada2 package (v3.13)^13^. Sequences were filtered and trimmed using the parameters: minQ = 3, minLen = 1000, maxLen = 2100, maxN = 0, rm.phix = FALSE, and maxEE = 2, followed by dereplication. Error rates were inferred using PacBioErrfun (errorEstimationFunction = PacBioErrfun) with randomize = TRUE, BAND_SIZE = 32, and multithread = TRUE. ASVs were then inferred using the pseudo-pooling strategy. Taxonomic assignment at the species level was performed, where possible, using the BLAST-based approach. Higher-rank classifications were obtained using the RDP classifier with a minimum bootstrap confidence threshold of 80%. ASVs lacking species-level annotation were further aligned against the previously generated OTU set using usearch_global with a 97% identity cutoff. The resulting ASV table underwent manual curation. ASVs were excluded if the RDP classifier identified them as chloroplast, eukaryotic, or archaeal sequences; if they lacked classification at the class level and did not match an OTU; if they mapped to OTUs previously removed during pipeline-based filtering; or if their assigned species or OTU had been flagged as a contaminant. Additionally, ASVs that could not be linked to either a species or an OTU were removed to further minimize potential contamination.

## RNA sequencing and analysis

Eukaryotic cytoplasmic and mitochondrial ribosomal RNA, along with bacterial ribosomal RNA were removed using specific depletion probes and magnetic beads provided in the Ribo-Zero Kit Epidemiology (Illumina, San Diego, CA, USA). This step enhances the representation of messenger RNA (mRNA) in the sample by reducing the overwhelming abundance of ribosomal RNA, which typically constitutes over 90% of total RNA. The total RNA, enriched mRNA and cDNA were assessed using the 2100 Bioanalyzer system with RNA 6000 Pico and High Sensitivity DNA kits (Agilent Technologies Inc, Santa Clara, USA). Libraries were generated using the ScriptSeq v2 RNA-Seq (Illumina), which enables directional (strand-specific) library construction, preserving transcriptional orientation. Sequencing was performed in single-end mode (50 or 68 base pairs) on an Illumina HiSeq 2500 platform using the TruSeq SBS Kit v3—HS (Illumina).

BCL files were converted to FASTQ files (single-end reads) using the bcl2fastq conversion software version v2.20.0.422 (Illumina). Quality control was performed with fastp, a FASTQ preprocessor^14^. To distinguish human from non-human reads, we utilized a mapping approach incorporating bwa-samse (bwa-0.7.17, build 0.7.17-r1188^15^) aligning the reads to the human reference genome sequence GRCh38.p13 (GCA_000001405.28), as provided by the NCBI RefSeq, PRJNA31257, currently maintained by the Genome Reference Consortium. Instead of discarding the human-aligned reads, they were separated and retained for downstream analysis of host gene expression. Only unmapped reads were retained for downstream microbial or metatranscriptomic analyses, ensuring high specificity in non-human signal detection.

To construct a metagenomic reference set tailored to the human oral cavity, sequences from two publicly available datasets were selected and integrated into a single comprehensive database. The first source was the expanded Human Oral Microbiome Database (eHOMD, Rev: 2021-10-01, full eHOMD contig set), a well-established resource in the field of oral metagenome analysis, which includes 2087 bacterial genomes from the human mouth and aerodigestive tract^16^. The second dataset originated from the study of Pasolli *et al.* 2019^17^, comprising 9,428 metagenomes and 154,723 metagenome-assembled genomes (MAGs) grouped into species-level genome bins (SGBs). From this source, the 7897 MAGs found in the study’s oral-cavity-derived samples were selected. The sequences from both sources were combined into a combined oral metagenome reference set, grouped into 95% average nucleotide identity (ANI) taxonomic units (TUs) representing the species level, and annotated with the Prokka pipeline^18^. To extend the annotation and to assign additional EC numbers for further analysis, the eggnog annotated orthology database was applied^19^. The original taxonomy provided by eHOMD, along with Prokka and eggNOG annotations, together constituted the functional and taxonomic framework of the reference set. To ensure relevance to the study cohort, only TUs with sufficient coverage by sequencing reads from the collected oral metagenome samples were retained, determined through mapping using BWA-samse ^16^^,^ ^20^. For the final mapping of the sample reads, all rRNA-coding genes were excluded from the reference based on the associated annotations.

To quantify transcript abundance, sample reads were mapped to the protein-coding gene sequences from the selected TUs (SGBs) within the customized human oral reference set using BWA-samse. To avoid a biased counting for the transcript abundance of features, the reads mapped to multiple features were randomly distributed between them during the mapping of the sample reads against the customized human oral bacteria reference. Read counts were then extracted from the aligned sequencing read files using the htseq-count program from the HTseq package^21^. To streamline analysis and emphasize functional interpretation, the counts were aggregated based on their associated EC numbers. For genes annotated with multiple EC numbers, counts were summed at the highest hierarchical EC level where annotations were consistent. If a gene’s EC annotations diverged at the second-highest hierarchical level, the corresponding read counts were categorized as "Unknown."

Taxonomic assignment of transcripts grouped by EC was performed following a method adapted from a previously published metatranscriptomic study^22^. Unlike the Jorth study, which reported species-level predictions, we opted to report genus- and class-level predictions to enhance accuracy. Briefly, read counts per locus were pre-filtered to include only the 1 million most abundant genes, in order to focus on biologically relevant transcripts and reduce noise from low-expression features. Hypothetical annotations were deselected and only genes with EC numbers were selected. For each sample, the counts per locus were normalized by the sum of read counts for all loci. Based on the functional annotation and taxonomic information of the individual gene, counts were then aggregated by the combination of EC number and taxon.

For the anabolic/catabolic comparison, the top 100 LDA-based ECs associated with health and peri-implantitis were selected and classified as anabolic, catabolic, or ambiguous using curated KEGG-based annotations. Briefly, ECs were categorized using a multi-step KEGG-based curation approach. EC numbers were first mapped to KEGG pathways and provisionally classified based on the KEGG pathway title and pathway category. Pathways explicitly labelled as biosynthesis or involved in biomass assembly including pathways labeled “biosynthesis,” fatty acid elongation, carbon fixation, and aminoacyl-tRNA biosynthesis were provisionally classified as anabolic, whereas pathways explicitly labelled as degradation, fermentation, oxidative phosphorylation or energy-yielding metabolism were provisionally classified as catabolic. Because individual ECs can occur in multiple KEGG pathways, provisional assignments were manually reviewed using KEGG EC names, reaction descriptions, and the specific KEGG pathways in which each EC occurred. Final categories were assigned only when the pathway annotation and enzyme function consistently supported a net anabolic or catabolic role. ECs with conflicting pathway assignments, reversible reactions, broad “metabolism” annotations, or unclear biological direction including central metabolism and interconversion pathways such as glycolysis/gluconeogenesis, pentose phosphate, pyruvate metabolism, and general amino-acid metabolism maps were classified as ambiguous. Category-level scores were computed by summing relative abundances within each group and expressing them as the log_2_(catabolism/anabolism) ratio, which served as the primary metric to account for compositional constraints and avoid redundancy. Differences between peri-implant health and peri-implantitis were evaluated using linear mixed-effects models, with the same model structure applied for the aerotolerant-to-anaerobic comparisons. Differences in the distribution of anabolic and catabolic EC categories between health- and peri-implantitis-associated enzymes were assessed using Fisher’s exact test.

We assessed abundance‐normalized taxon activity by pairing full-length 16S (DNA) and metatranscriptomic (RNA) relative abundances at the level of taxonomic classes and computing log2(RNA/DNA) for each taxon in each sample, following the RNA/DNA log-ratio framework described by Franzosa et al ^23^.   To stabilize ratio estimates in the presence of low-frequency observations/zeros, we applied sample-wise adaptive smoothing prior to ratio calculation. Deviation of log2(RNA/DNA) from zero was evaluated using one-sample t-tests with Benjamini–Hochberg FDR correction.

A total of 48,905 human-associated phage genomes from the oral cavity were selected from the IMG/VR database (IMG_VR_2018-07-01_4 – IMG/VR v2)^24^, based on metadata information in the IMGVR_all_Sequence_information.tsv file. These comprised genomes from cultivated viral isolates (iVGs), uncultivated viral genomes (UViGs), and prophages. These genomes were downloaded, indexed, and used as a reference for mapping RNA-seq reads using Bowtie2 (v2.3.5.1) with default settings. Read counts per genome per sample were used for the downstream multivariate analysis. Reads were aggregated either at the viral Operational Taxonomic Unit (vOTU) level or according to predicted phage hosts at the genus level. Viral host assignments were retrieved from IMG/VR v2^24^. Briefly, host predictions were based on: (i) documented host information for iVGs; (ii) curated prophage–host associations^25^; and (iii) sequence matches between viral contigs and microbial CRISPR-Cas spacer arrays^24^.

To investigate phage-bacterium relationships, we applied two complementary analytical strategies. First, we correlated clusters of bacterial species/OTUs derived from Full-16S (DNA-based) with clusters of phage species/vOTUs identified by MTX (RNA-based) using hierarchical all-against-all association testing (HAllA)^26^. We then evaluated whether bacterial species associated with specific phage clusters corresponded to their predicted hosts according to IMG-VR annotations. In other words, we assessed whether phage transcriptional activity correlated with the abundance of their predicted hosts at high taxonomic resolution (species level). Second, to complement the species-level analysis and reduce feature dimensionality, we aggregated bacterial taxa at the genus level and grouped phages according to their predicted host genus. This broader framework utilized hierarchical clustering and enabled evaluation of host–phage concordance at a higher taxonomic rank.

Representative genomes of the most abundant peri-implant phage species/vOTUs were re-analyzed using the PhaBOX web server (v2) for characterization of phage contigs^27^. Specifically, PhaMer was used for phage identification^28^, PhaTYP for lifestyle prediction^29^, PhaGCN for taxonomic classification^30^, and CHERRY for host prediction^31^. All databases were curated to conform to the ICTV 2024 taxonomy release^32^. For each species/vOTU (n = 5; vOTU_020193, vOTU_000716, vOTU_002684, vOTU_000615, vOTU_000953), the IMG/VR high quality draft genome with the highest estimated completeness of 99% was selected as the representative sequence (IMG/VR vOTUs were defined by single-linkage clustering of sequences sharing ≥95% average nucleotide identity across ≥85% of the length of the shorter sequence^24^). In cases where completeness metrics were unavailable (n = 4; vOTU_004272, vOTU_011788, sg_290291, vOTU_098376), the IMG/VR longest assembled genome fragment (ranging 13102 – 45798 nt) was used as a proxy.

For the human transcriptome analysis, the latest annotated version of the human reference genome GRCh38.p13 (GCA_000001405.28), which includes 63,925 genes, was used as the reference. Samples with fewer than 500,000 reads mapped to human reference genome were excluded. To normalize for sequencing depth, read counts were standardized within each sample. Only the top 2,000 genes, accounting for ~70% of total reads on average, were retained for downstream analysis.

**Scanning electron microscopic characterization of submucosal biofilm fragments from peri-implantitis lesions**

Glass slides (CB00120RAC, Thermo Fisher) were sterilized under UV light for 30 minutes, coated with Poly-D-Lysine, and incubated for 1 hour at room temperature. Unbound material was then removed, and the preconditioned slides were used for SEM analysis. A volume of 35–50 µl of biofilm sediment in reduced transport fluid, representing suspensions of clinical peri-implant biofilm fragments collected by curette, was pipetted onto the prepared slides and incubated for 1 hour at room temperature in 24-well plate. Excess liquid was carefully removed with a pipette. Samples were fixed in 500 µl of 1.5% paraformaldehyde and 1.5% glutaraldehyde in 0.15 M HEPES Buffer. Samples were stored at 4 °C until further processing. For dehydration, bacterial cells were subjected to an ascending acetone series, followed by critical-point-drying. Finally, samples were sputter-coated with gold and examined using a scanning electron microscope (Crossbeam540, Zeiss) operated at 10 kV.

# Supplemental Tables and Figures

**Supplemental Table 1 ǀ Patient and implant characteristics.** Patient and implant characteristics were compared across three diagnosis groups: health (PIH) , mucositis (PIM) , and peri-implantitis (PI).

Supplementary Tables 2-15 (listed below) are available in the separate file *Supplemental_Data_Files_1_of_2.xlsx* (2-9) and *Supplemental_Data_Files_2_of_2.xlsx* (10-15)*.*

**Supplemental Table 2 ǀ Overview of full-length 16S rRNA gene reads and RNAseq reads per sample.**

**Supplemental Table 3 ǀ Class-level taxonomic profiles across diagnosis groups.** Relative abundances of genera, statistical comparisons between clinical groups (Kruskal-Wallis P-values), and LDA effect sizes from CAP analysis.

**Supplemental Table 4 ǀ Genus-level taxonomic profiles across diagnosis groups.** Relative abundances of genera, statistical comparisons between clinical groups (Kruskal-Wallis P-values), and LDA effect sizes from CAP analysis.

**Supplemental Table 5 ǀ Species-level taxonomic profiles across diagnosis groups.** Relative abundances of genera, statistical comparisons between clinical groups (Kruskal-Wallis P-values), and LDA effect sizes from CAP analysis.

**Supplemental Table 6 ǀ Class-level RNA:DNA abundance ratios (log2) across samples with *p* values**

**Supplemental Table 7 ǀ Differential expression of metatranscriptomic ECs across diagnosis groups.**

**Supplemental Table 8 ǀ Differential expression of ECs across microbial community types (CTs).**

**Supplemental Table 9 ǀ Average relative expression levels of functional ECs stratified by contributing taxa.**

**Supplemental Table 10 ǀ Ecological roles and metabolic associations of top 60 highly expressed ECs across diagnosis groups and CTs.**

**Supplemental Table 11 ǀ Differential expression of phage-derived transcripts across diagnosis groups.**

**Supplemental Table 12 ǀ Associations identified by HAllA (Hierarchical All-against-All) analysis between taxonomic species (16S data) and functional EC numbers (MTX data).**

**Supplemental Table 13 ǀ Associations identified by HAllA (Hierarchical All-against-All) analysis between taxonomic species (16S data) and phage transcripts profiles (MTX data).**

**Supplemental Table 14 ǀ Associations identified by HAllA (Hierarchical All-against-All) analysis between taxonomic species (16S data) and host genes transcripts (MTX data).**

**Supplemental Table 15 ǀ Associations identified by HAllA (Hierarchical All-against-All) analysis between functional EC numbers (MTX data) and host genes transcripts (MTX data).**

**Supplemental Figure 1. Bacterial RNA yield across diagnostic groups and plaque index scores.** **a**, Diagnostic groups. Violin plots show the distribution of extracted bacterial RNA (ng) for health (PIH), mucositis (PIM), and peri-implantitis (PI) samples, with individual data points overlaid (jittered) and summarized as boxplots (box: 25th–75th percentile; whiskers: 1.5× IQR; center line: median). The y-axis is log10-scaled to accommodate the wide range of RNA yields (0.05–1453.7 ng). Groups were compared using the Kruskal-Wallis test (H = 24.5, *P* = 4.7 ∙ 10^-6^) with Dunn's post-hoc correction for multiple comparisons (PIH vs. PIM, *P* = 0.0096; PIM vs. PI, *P* = 0.04; PIH vs. PI, *P* = 2.1 ∙ 10^-6^). **b**, Plaque index scores. The same data as in **a** are shown, stratified by plaque index score and colored by diagnostic group. Kruskal-Wallis P < 0.0001; Spearman ρ = 0.49 (*P* = 1.75∙10^-8^; 95% CI 0.34-0.62).

**Supplemental Figure 2. Biofilm morphology.** Scanning electron micrographs of submucosal implant-associated biofilms from peri-implantitis. Main components of biofilms are highlighted.

**Supplemental Figure 3. Four active community types.** **a**, General characteristics. Taxonomy at class level, diagnosis and four clinical measures are plotted for each community type. **b** – **d**, ordinations representing peri-implant samples overlaid with SIMPROF clusters, diagnosis, as well as classes and clinical measures as vectors, respectively. Non-metric multidimensional scaling (MDS) was used to represent the peri-implant samples in two-dimensional space.

**Supplemental Figure 4. *Neisseria* abundance, species composition, and community diversity across diagnosis groups.**

**a**, Genus-level *Neisseria* relative abundance across peri-implant health (PIH), peri-implant mucositis (PIM), and peri-implantitis (PI), shown as log(x+1)-transformed relative abundances. Each dot represents one implant sample. **b**, Composition of different *Neisseria* species across diagnosis groups. **c**, Heatmap of log(x+1) transformed abundances for *Neisseria* amplicon sequence variants (ASVs) grouped by species across individual samples, ordered by diagnosis. **d**, alpha diversity of Neisseria ASVs across diagnosis groups shown as richness (S), Shannon diversity (H′), and Simpson diversity (1-Lambda), displayed as boxplots with individual samples shown as dots.

**Supplemental Figure 5. Correlation of the Full-16S and RNA based relative abundances of bacterial classes. a**, Paired DNA-RNA scatterplots showing, for each bacterial class, the relationship between DNA (full-length 16S) relative abundance and RNA (metatranscriptomic) relative abundance across samples, coloured by diagnosis groups. Axes are shown on a log scale. b, Heatmap of abundance-normalized activity computed as log_2_(RNA/DNA) for each class in each sample, with samples ordered by diagnosis group. Values are capped at ±2 for visualization; 0 indicates RNA proportional to DNA abundance, positive values indicate RNA enrichment relative to DNA (greater transcript contribution than expected from abundance), and negative values indicate RNA depletion. Rows are hierarchically clustered using 1 − Spearman correlation distance (average linkage). The panel on the right shows the median log_2_(RNA/DNA) within each diagnosis group using the same row order as the per-sample heatmap.

**Supplemental Figure 6. EC activities across diagnosis groups. a**, Unconstrained ordination for active ECs expression data. MDS plot calculated for X implants on Bray-Curtis dissimilarities for log(x+1)-transformed read counts grouped to ECs (relative activity cut-off = 0.1%). **b**, Heatmap for ECs. First, samples and ECs were each clustered, and next samples were sorted by diagnosis. **c**, Diagnosis-constrained ordination for ECs data. CAP plot calculated on same input data as for “a”. Selected diagnosis-correlated ECs ( ǀ r ǀ > 0.6) in CAP are indicated as vectors.

**Supplemental Figure 7. Active phageome across diagnosis groups.** **a**, Unconstrained ordination for active bacteriophage species (phage species, for short) expression data. MDS plot calculated for 88 implants on Bray-Curtis dissimilarities for log(x+1)-transformed read counts grouped to phage species (relative activity cut-off = 0.1%). **b**, Heatmap for phage species. First, samples and phage species were each clustered, and next samples were sorted by diagnosis. **c**, Diagnosis-constrained ordination for phage species data. CAP plot calculated on same input data as for “a”. Selected diagnosis-correlated phage species ( ǀ r ǀ > 0.6) in CAP are indicated as vectors.

**Supplemental Figure 8. Host gene expression across diagnosis groups. a**, Unconstrained ordination for host gene expression data. MDS plot calculated for 91 implants on Bray-Curtis dissimilarities for log (x+1)-transformed read counts grouped to highly expressed host genes (relative activity cut-off = 0.1%). **b**, Heatmap for highly expressed genes. First, samples and genes were each clustered, and next samples were sorted by diagnosis. **c**, Diagnosis-constrained ordination for host gene expression data. CAP plot calculated on same input data as for “a”. Selected diagnosis-correlated genes ( ǀ r ǀ > 0.6) in CAP are indicated as vectors.

# References

1.	Berglundh, T. et al. Peri-implant diseases and conditions: Consensus report of workgroup 4 of the 2017 World Workshop on the Classification of Periodontal and Peri-Implant Diseases and Conditions. *Journal of Clinical Periodontology* **45**, S286-S291 (2018).

2.	Szafrański, S.P. et al. Functional biomarkers for chronic periodontitis and insights into the roles of Prevotella nigrescens and Fusobacterium nucleatum; a metatranscriptome analysis. *npj Biofilms and Microbiomes* **1**, 15017 (2015).

3.	Grischke, J., Szafrański, S.P., Muthukumarasamy, U., Haeussler, S. & Stiesch, M. Removable denture is a risk indicator for peri-implantitis and facilitates expansion of specific periodontopathogens: a cross-sectional study. *BMC Oral Health* **21**, 173 (2021).

4.	Dieckow, S. et al. Structure and composition of early biofilms formed on dental implants are complex, diverse, subject-specific and dynamic. *npj Biofilms and Microbiomes* **10**, 155 (2024).

5.	Desch, A. et al. Biofilm formation on zirconia and titanium over time-An in vivo model study. *Clinical oral implants research* **31**, 865-880 (2020).

6.	Quast, C. et al. The SILVA ribosomal RNA gene database project: improved data processing and web-based tools. *Nucleic acids research* **41**, D590-596 (2013).

7.	Chen, T. et al. The Human Oral Microbiome Database: a web accessible resource for investigating oral microbe taxonomic and genomic information. *Database : the journal of biological databases and curation* **2010**, baq013 (2010).

8.	Yarza, P. et al. The All-Species Living Tree project: a 16S rRNA-based phylogenetic tree of all sequenced type strains. *Systematic and applied microbiology* **31**, 241-250 (2008).

9.	Edgar, R.C. UPARSE: highly accurate OTU sequences from microbial amplicon reads. *Nature methods* **10**, 996-998 (2013).

10.	Wang, Q., Garrity, G.M., Tiedje, J.M. & Cole, J.R. Naive Bayesian classifier for rapid assignment of rRNA sequences into the new bacterial taxonomy. *Applied and environmental microbiology* **73**, 5261-5267 (2007).

11.	Salter, S.J. et al. Reagent and laboratory contamination can critically impact sequence-based microbiome analyses. *BMC Biology* **12**, 87 (2014).

12.	Szafranski, S.P. et al. High-resolution taxonomic profiling of the subgingival microbiome for biomarker discovery and periodontitis diagnosis. *Applied and environmental microbiology* **81**, 1047-1058 (2015).

13.	Callahan, B.J. et al. DADA2: High-resolution sample inference from Illumina amplicon data. *Nature methods* **13**, 581-583 (2016).

14.	Chen, S., Zhou, Y., Chen, Y. & Gu, J. fastp: an ultra-fast all-in-one FASTQ preprocessor. *Bioinformatics* **34**, i884-i890 (2018).

15.	Li, H. & Durbin, R. Fast and accurate short read alignment with Burrows-Wheeler transform. *Bioinformatics* **25**, 1754-1760 (2009).

16.	Escapa, I.F. et al. New Insights into Human Nostril Microbiome from the Expanded Human Oral Microbiome Database (eHOMD): a Resource for the Microbiome of the Human Aerodigestive Tract. *mSystems* **3** (2018).

17.	Pasolli, E. et al. Extensive Unexplored Human Microbiome Diversity Revealed by Over 150,000 Genomes from Metagenomes Spanning Age, Geography, and Lifestyle. *Cell* **176**, 649-662 e620 (2019).

18.	Seemann, T. Prokka: rapid prokaryotic genome annotation. *Bioinformatics* **30**, 2068-2069 (2014).

19.	Huerta-Cepas, J. et al. eggNOG 5.0: a hierarchical, functionally and phylogenetically annotated orthology resource based on 5090 organisms and 2502 viruses. *Nucleic acids research* **47**, D309-D314 (2019).

20.	Danecek, P. et al. Twelve years of SAMtools and BCFtools. *GigaScience* **10** (2021).

21.	Anders, S., Pyl, P.T. & Huber, W. HTSeq--a Python framework to work with high-throughput sequencing data. *Bioinformatics* **31**, 166-169 (2015).

22.	Jorth, P. et al. Metatranscriptomics of the human oral microbiome during health and disease. *mBio* **5**, e01012-01014 (2014).

23.	Franzosa, E.A. et al. Relating the metatranscriptome and metagenome of the human gut. *Proceedings of the National Academy of Sciences of the United States of America* **111**, E2329-2338 (2014).

24.	Paez-Espino, D. et al. IMG/VR v.2.0: an integrated data management and analysis system for cultivated and environmental viral genomes. *Nucleic acids research* **47**, D678-D686 (2019).

25.	Roux, S., Hallam, S.J., Woyke, T. & Sullivan, M.B. Viral dark matter and virus–host interactions resolved from publicly available microbial genomes. *eLife* **4**, e08490 (2015).

26.	Ghazi, A.R. et al. High-sensitivity pattern discovery in large, paired multiomic datasets. *Bioinformatics* **38**, i378-i385 (2022).

27.	Shang, J., Peng, C., Liao, H., Tang, X. & Sun, Y. PhaBOX: a web server for identifying and characterizing phage contigs in metagenomic data. *Bioinformatics advances* **3**, vbad101 (2023).

28.	Shang, J., Tang, X., Guo, R. & Sun, Y. Accurate identification of bacteriophages from metagenomic data using Transformer. *Briefings in Bioinformatics* **23** (2022).

29.	Shang, J., Tang, X. & Sun, Y. PhaTYP: predicting the lifestyle for bacteriophages using BERT. *Briefings in Bioinformatics* **24** (2022).

30.	Shang, J. & Sun, Y. Predicting the hosts of prokaryotic viruses using GCN-based semi-supervised learning. *BMC Biology* **19**, 250 (2021).

31.	Shang, J. & Sun, Y. CHERRY: a Computational metHod for accuratE pRediction of virus–pRokarYotic interactions using a graph encoder–decoder model. *Briefings in Bioinformatics* **23** (2022).

32.	Simmonds, P. et al. Changes to virus taxonomy and the ICTV Statutes ratified by the International Committee on Taxonomy of Viruses (2024). *Archives of Virology* **169**, 236 (2024).
